# Current status and future prospects of microalgae-based degradation of spent lubricant oil hydrocarbon towards environmental sustainability: a mini review and bibliometric analysis

**DOI:** 10.1007/s00203-025-04332-0

**Published:** 2025-05-19

**Authors:** Stella B. Eregie, Isaac A. Sanusi, Olaniran O. Ademola

**Affiliations:** 1 School of Life Sciences, University of KwaZulu-Natal, Private Bag, X01, Pietermaritzburg, 3209 South Africa; 2 School of Life Sciences, University of KwaZulu-Natal, Private Bag X54001, Westville, South Africa

**Keywords:** Spent oil waste, Microalgae: biodegradation: environment: bibliometric

## Abstract

The biodegradation of spent oil waste (SOW) using bacteria and fungi has been actively researched over the years. Only recently has the use of microalgae for the treatment of SOW attracted significant attention. This review aims to highlight the biodegradative capabilities of microalgae as well as provide a comprehensive bibliometric analysis to assess current research activities and trends in microalgae-based biodegradation of SOW. The bibliographic data exported from Dimensions database was analyzed using VOSviewer, focusing on various aspects such as document types, publications, subject categories, sources, countries, authors, organizations, and cited articles. The results obtained showed a remarkable increase (80.23%) in the number of article publications from 2005 to 2023 in this field of research. China (887 publications), Environmental Science (3571 publications), Bioresource Technology (249 publications) and Harbin Institute of Technology (72 publications), were the most productive country, subject category, journal, and organization, respectively, publishing articles in this field of research. The review also discussed SOW hydrocarbons ranging from alkanes, aromatic compounds to polychlorinated compounds and the mechanism of degradation of these compounds by microalgae. Overall, the review provided useful insight on microalgae SOW degradation, current research direction and the prospect of using microalgae in environmental remediation and sustainability.

## Introduction

Hydrocarbon wastes such as spent oil waste (SOW), are generated from industrial processes, automotive engines, machinery, workshops, mechanical processes mining activities and agricultural activities (Soumeya et al. 2022). The generation of SOW has attracted global interest, particularly in nations with growing industrial activities and populations (Pongsilp and Nimnoi 2022). Global generation of SOW is steadily increasing each year (Medić et al. 2020), and according to Gertsen et al. (2023), SOW is the most difficult oil waste to be disposed, mainly because of its high chemical contents which are recalcitrant and toxic (Obi et al. 2022). In 2016, the global SOW generation reached 2.017 billion tons per year, and projections for 2050 indicated a 70% increase, reaching up to 3.4 billion tons per year (Kuttiyathil et al. 2021; Touliabah et al. 2022). In 2020, there was a notable rise of 0.9 million barrels per day in the production of SOW, while the demand for lubricant oil reached unprecedented levels, hitting 100 million barrels per day globally (Pongsilp and Nimnoi 2022). From recent reports, the total amount of SOW that goes into the environment each year globally is approximately 200 billion tons (Gertsen et al. 2023; Stepanova et al. 2022). Of this volume, only a small percentage is successfully treated via recycling or incineration. While, the major proportion of SOW enters the environment directly or indirectly, as effluents or spills, or by runoff or atmospheric deposition. As a result of increased industrial activities, the environment is being polluted with SOW from various sources. The ecological disaster caused from improper hydrocarbon such as SOW disposal to the ocean and coastline could kill thousands of ocean birds (Mapelli et al. 2017). In some cases, over 60% of aquatic creatures as well as affecting the nearby residential communities have been reported (Bovio et al. 2017). Clean-up due to hydrocarbon pollution was sometime valued around 10 billion dollars (Alessandrello et al. 2017; Mapelli et al. 2017).

The SOW contains a broad range of hydrocarbons (HCs), including aromatics such as monoaromatic hydrocarbons, polycyclic aromatic hydrocarbons (PAHs), and polychlorinated biphenyls (PCBs) known to be toxic and carcinogens (Touliabah et al. 2022). The accumulation and persistence of SOW environment poses a serious threat to humans, animals, plants, fresh water, and marine ecosystems (Touliabah et al. 2022; Davoodi et al. 2020). Various physiochemical treatment techniques such as landfilling, incineration and burying, evaporation, dispersion, excavation, solvent extraction, chemical decomposition, recycling, and composting have been employed to treat and reclaim SOW polluted site (Gertsen et al. 2023; Stepanova et al. 2022). These physiochemical treatments require high operational and maintenance cost (Pongsilp and Nimnoi 2022). These methods also produce secondary pollutants that require additional treatment with specialized equipment, as many of the hazardous pollutants are combustible, highly volatile, and extremely flammable. Similarly, solvent extraction and chemical decomposition is another method used for the pretreatment of SOW, but when solvent extraction and chemical decomposition of SOW is not properly managed (which could be challenging), can also release harmful pollutants into the atmosphere and discharge toxic chemicals into the water and land environments (Dell’Anno et al. 2021; Radziff et al. 2021). Exposure to organic solvents or chemicals poses significant chemical health risks (Pi et al. 2023; Vimali et al. 2021). SOW in water produces an oily film, which limit the inflow of oxygen thereby endangering aquatic species (Sattar et al. 2022). On the other hand, when SOW leaches into the soil, plant roots have difficulty in absorbing and respiring oxygen, leading to death (Obi et al. 2022; Sattar et al. 2022). Ingesting the undegraded SOW endangers human and animal health causing irritation, neurodegenerative diseases, bone marrow damage, and the risk of various type of cancer (Obi et al. 2022; Sattar et al. 2022). In the light of the various physiochemical treatment limitations, there is an urgent need to develop sustainable, eco-friendly, and cost-effective solutions, such as bio-based solutions to reduce the harmful effects of the SOW contaminants. Reports on bio-based solutions have been limited, efforts such as current review update on bio-based solutions in this regard are being encouraged. Such review will give update on latest research trends and niche.

**Fig. 1:**
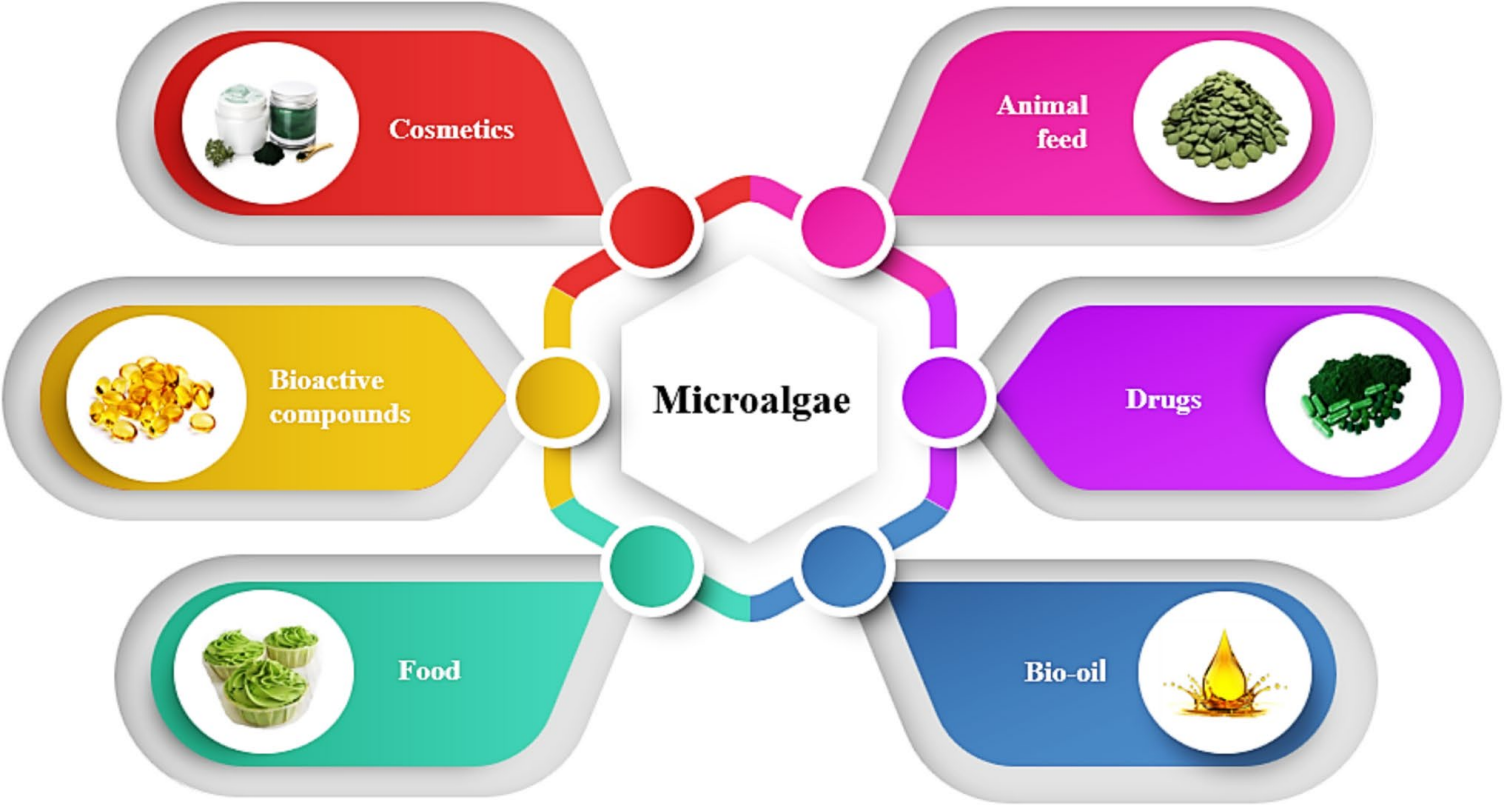
Biotechnological application of microalgae

The use of microalgae as one of the bio-based approach have sparked a lot of scientific interest in recent years as an economic and eco-friendly technology for treating spent oil polluted areas compared to physiochemical treatment techniques (Touliabah et al. 2022). This is because of their (1) cost effectiveness, (2) environmental friendliness, (3) high adaptability, biosorption and tolerance to organic pollutants, (4) low energy usage, (5) lower risk of environmental contamination, and (6) degradation efficiency (Al-Hussieny et al. 2020; Dell’Anno et al. 2021; Touliabah et al. 2022). Microalgae represent an under explored resource, with an estimated species number of 30,000 to 1,000,000 (Rumin et al. 2020). Figure 1 depicts the diverse applications of microalgae, showing their potentials in various industries (Gonçalves 2021; Ismail et al. 2024).

Over the past few years, several microalgae species have been successfully utilized in biodegradation of pollutants found in industrial SOW (details on specific hydrocarbons and corresponding microalgae involved in their degradation are elucidated later in the study). Microalgae species in this regard include the general *Selenastrum, Scenedesmus, Chlorella,* and *Chlamydomonas* species. This is owning to their unique physiological and biochemical traits, high growth rate as well as possession of unique catabolic genes (Aldaby and Mawad 2019; Dell’Anno et al. 2021; Radziff et al. 2021). These microalgae species degrade HCs by adsorption of the HCs on the cell surface, then the HCs is bioaccumulated and emulsified within the cells and ingested via endocytosis (either by active transport or passive transport) biotransforming the HCs into harmless compounds, such as carbon dioxide and water (Sutherland and Ralph 2019). Despite the growing interest in utilizing microalgae in the biodegradation of SOW, comprehensive bibliometric analyses in this area remain limited. Bibliometric analysis will provide insights on scientific efforts and emerging trends in this research area.

The bibliometric analysis is a statistical approach that has been employed to analyze scientific efforts and emerging trends in different research field such as bibliometric reviews in microalgae research (Eregie et al. 2025; Purba et al. 2024; Loh et al. 2023; Melo et al. 2022; Ubando et al. 2021). For instance, Ubando et al. (2021) conducted a bibliographic review on microalgal biosorption of heavy metals. Similarly, Purba et al. (2024) and Melo et al. (2022) carried out bibliometric review of microalgae cultivation in industrial wastewater and agricultural wastewater. To date, there is a dearth of report on comprehensive bibliometric analysis of microalgae in treating SOW. Various databases, including Scopus, Web of Science, and Google Scholar have reported on oil waste treatment using microalgae. These databases are subscription-based, have restricted assess, and primarily focus on peer-reviewed literature. Due to these limitations, its crucial to find alternative database. In this context, Dimensions has emerged as a potential substitute to subscription-based databases such as Scopus, Web of Science, and Google Scholar. It offers several advantages: (1) access to a wider range of research outputs, including preprints, datasets, grants, clinical trials, patents, reports, and journal articles, (2) free access and more open access options, (3) cost-effectiveness, (4) flexible searching capabilities, and (5) access to a broader array of disciplines and journals (Hakkaraki 2024). Using Dimensions as a data mining source for microalgae in SOW treatment could be a noteworthy approach to identify recent scientific publications and analyze global research trends in this specific area. This approach could offer valuable insights into the broader scope of microalgae-based technology and future research prospects, providing a useful resource for scholars interested in this field. By providing insights on research productivity of authors, research groups, journals, institutions, countries and collaboration between them, thereby contributing to a better understanding of the current state and future potential in this research niche. Presently, there is scarcity of report on research productivity of authors, researcher group, institution and countries in regard to microalgae-based biodegradation of SOW. This review highlights the biodegradative capabilities of microalgae, elucidate the global research activities as well as trends on microalgae-based degradation of SOW via bibliometric analysis. It also discusses hydrocarbons in SOW, their environmental impacts and the major mechanism of hydrocarbon degradation by microalgae.

## Bibliometric review methodology

### Data collection

**Fig. 2:**
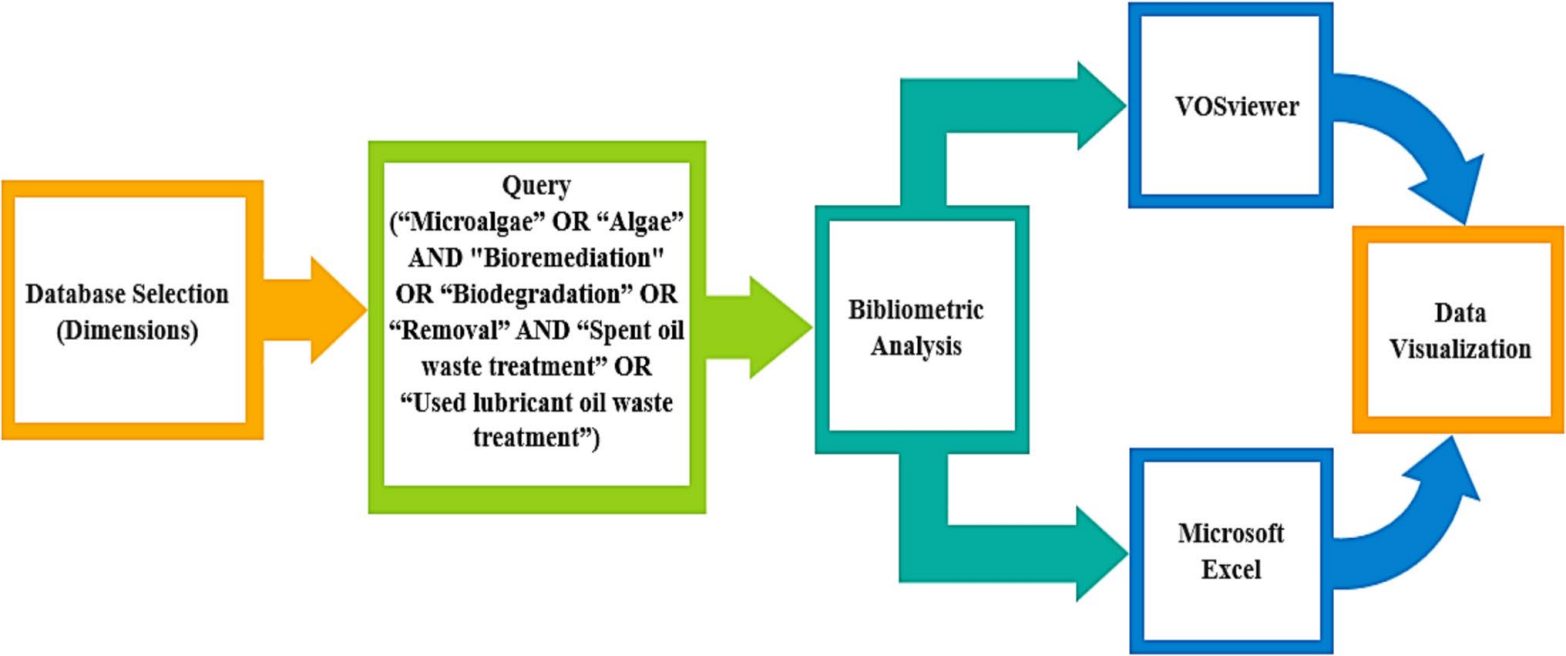
The schematic representation of the bibliometric analysis

The schematic representation of the bibliometric workflow in retrieving the information about the research topic is shown in Fig. 2. The bibliographic data was obtained using the Dimensions database September 18, 2024. The search and data collection involves three phases: an extensive literature search, in-depth screening and extraction of the relevance data and a bibliometric analysis. The different combinations of keywords search utilized in the study are listed in Table 1. The keyword selection process was refined to ensure relevance to the research topic. To refine the search, a combination of broad and relevant keywords, along with synonyms, abbreviations, and related terms, were utilized. To further refine the search results, Boolean operators such as “AND” and “OR” were applied to the search to narrow or broaden the results. Based on the results, search terms were adjusted to exclude irrelevant keywords and incorporate more specific phrases. Amongst the various words’ combinations, the category number 14 composed of “Microalgae” OR “Algae” AND “Bioremediation” OR “Biodegradation” OR “Removal” AND “Spent oil waste treatment” OR “Used lubricant oil waste treatment” have the highest total number of documents in terms of articles, book chapters and proceedings compared to other keywords combinations. The bibliometric analysis was performed solely using category numbers 14. A total number of 7,785 documents published from 1882 to 2023 were obtained from Dimensions database after search refinement with the category number 14. The exported bibliographic data in terms of country affiliation of publications, most productive journals, authors, organizations, and most cited documents were analyzed and visualized based on a threshold of three published articles, citations and total link strength using the VOSviewer software version 1.6.20 and Microsoft Excel 2019, Office 365 version 16.0.

**Table 1:** Total number of documents from Dimensions keyword search (September 18, 2024)

Category	Words search	Number of articles
1	“Microalgae” AND “Spent oil waste treatment”	0
2	“Microalgae” AND “Spent oil waste” AND “Biodegradation”	5
3	“Microalgae” AND “Spent oil waste” AND “Bioremediation”	5
4	“Microalgae” AND “Spent oil waste”	6
5	Microalgae” AND “Spent oil waste” AND “Treatment”	6
6	“Microalgae” AND “Spent oil waste” AND “Industrial waste”	2
7	“Microalgae” AND “Spent oil waste” AND “Hazardous waste”	2
8	“Microalgae” AND “Spent oil waste” AND “pollutants”	5
9	“Microalgae” AND “Spent oil waste” AND “Hydrocarbon”	5
10	“Microalgae” OR “Algae” AND “Bioremediation” OR “Biodegradation” AND “Spent oil waste treatment” OR “Used lubricant oil waste treatment”	1,522
11	“Microalgae Biodegradation” AND “Spent oil waste treatment” OR “Used lubricant oil waste treatment”	149
12	“Microalgae” OR “Macroalgae” AND “Bioremediation” OR “Biodegradation” OR “Biotransformation” AND “Spent oil waste treatment” OR “Used lubricant oil waste treatment”	38
13	“Microalgae” OR “Algae” AND “Bioremediation” OR “Biodegradation” OR “Biotransformation” AND “Spent oil waste treatment” OR “Used lubricant oil waste treatment”	396
14	“Microalgae” OR “Algae” AND "Bioremediation" OR “Biodegradation” OR “Removal” AND “Spent oil waste treatment” OR “Used lubricant oil waste treatment”	7785

## Bibliometric results and discussion

### Analysis of document types

**Fig. 3:**
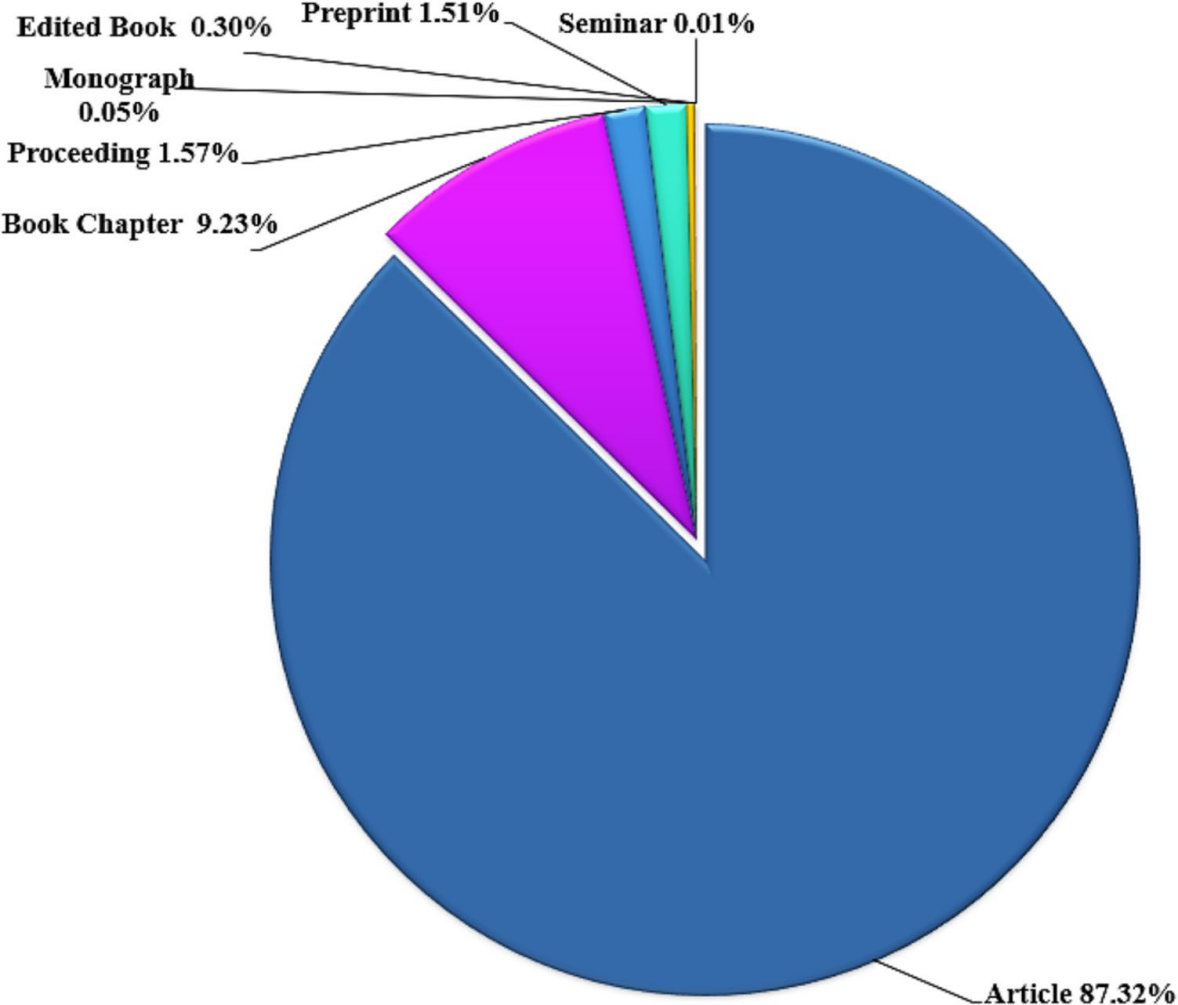
Types of documents in dimensions data database

Figure 3 shows the distribution of document types related to microalgae-based biodegradation of SOW. Articles formed the overwhelming majority (87.32%, 6,803 records), followed by chapters (9.23%, 719 records), proceedings (1.57%, 122 records), and preprints (1.51%, 118 records). The remaining categories, including edited books, monographs, and potentially miscategorized seminars, collectively represented less than 1% of the total documents. The notable increase in the number of articles over the last decade suggests a growing interest in this research area. The dominance of articles underscores the research evolution in this field. This also shows the researchers engagement in exploring innovative solutions for environmental challenges, as articles often present novel findings and methodologies, disseminate research outcomes, and facilitate the rapid transfer of knowledge (Bornmann and Mutz 2015).

### Analysis of trends in publication years

**Fig. 4:**
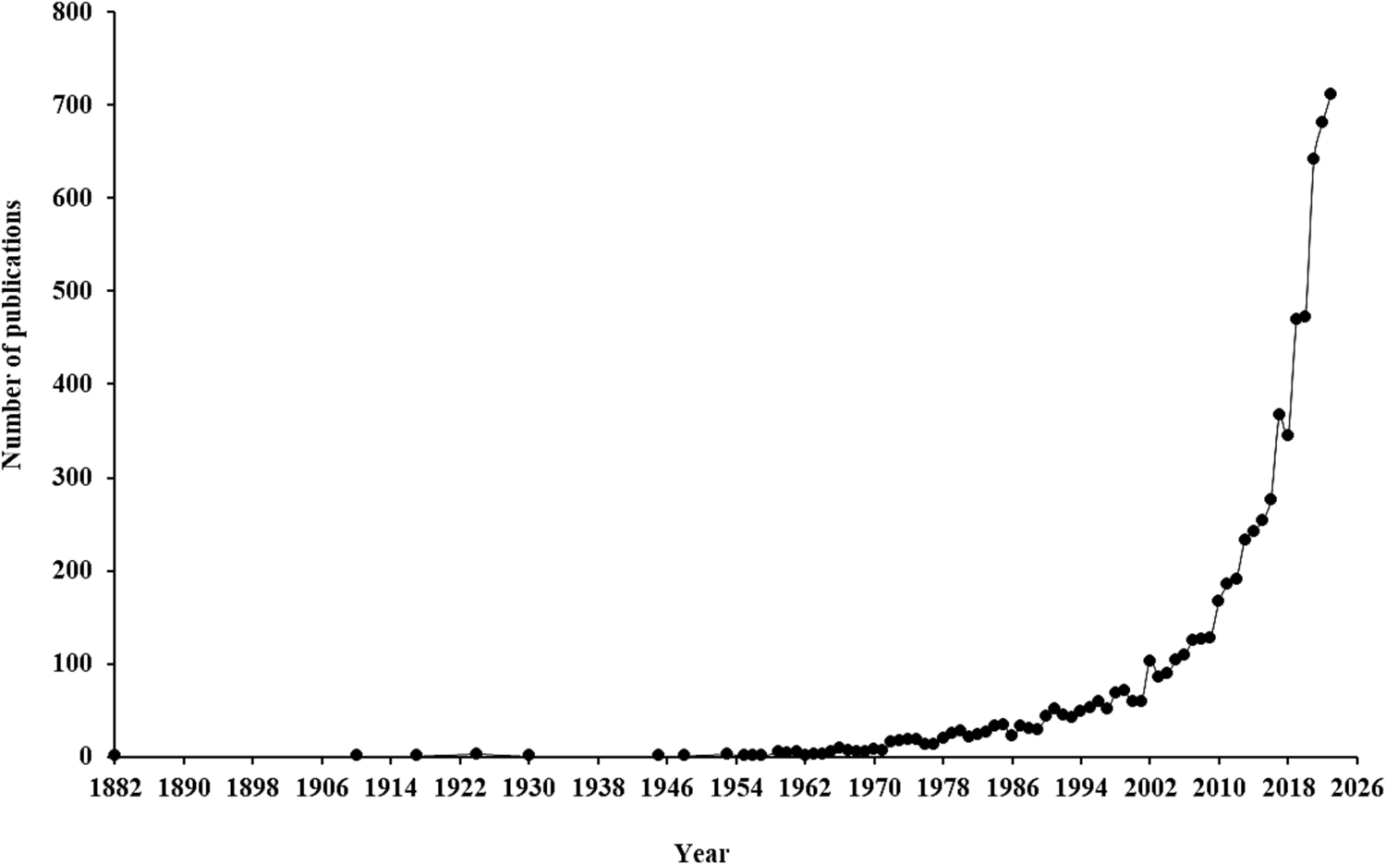
Scientific publication obtained from 1882 to 2023 with the combination of “microalgae” or “algae” and “bioremediation” or “biodegradation” or “removal” and “spent oil waste treatment”

A bibliometric analysis on microalgae-based biodegradation of SOW was carried out to evaluate the research activities, trends and growth or lack of research in this area. Figure 4 presents the scientific article published globally about microalgae-based biodegradation of SOW. From 1882 to 1977, the scientific paper publications were relatively low (less than 20 scientific papers were published globally) accounting for 2.50% of the total articles published (Fig. 4). The relatively low number of scientific papers observed from 1882 to 1977 indicates that this research field was in its infancy and of low scientific interest. This could possibly be due to lack of awareness, funding constraints, lack of technological know-how and standardized methodologies. A fluctuating trend in the number of articles published per year was observed between 1978 and 2004, accounting for 17.27% of the total articles published (Fig. 4), suggesting variability in research interest, advancements in scientific methods and global innovations in technologies. Conversely, analysis of publication from 2005 to 2023 showed a remarkable increase (80.23%) in the number of publications compared to publications obtained for 1882–1977 and 1978–2004) (Fig. 4). The substantial increase in the number of publications from 2005 to 2023 underscores a significant growing scientific interest and research activity, increased funding, advancement in methodologies, enhanced global collaboration and technological improvement. The increased number of publications also underscores the field’s critical role in addressing industrial, environmental, and global challenges, including public health concerns. This trend shows a global recognition of the field’s relevance and its expanding role in developing sustainable and impactful solutions. In line with the present review, Radziff et al. (2021) and Melo et al. (2022) using bibliometric analysis reported increase in scientific paper publications in microalgae-based research in the treatment of organic pollutants. Likewise, using bibliometric approach, Ubando et al. (2021) and De Souza et al. (2019) corroborated the submission that microalgae-based treatment as a green technology for the next generation bioremediation of contaminants is evolving rapidly.

### Analysis of publications in subject areas

**Fig. 5:**
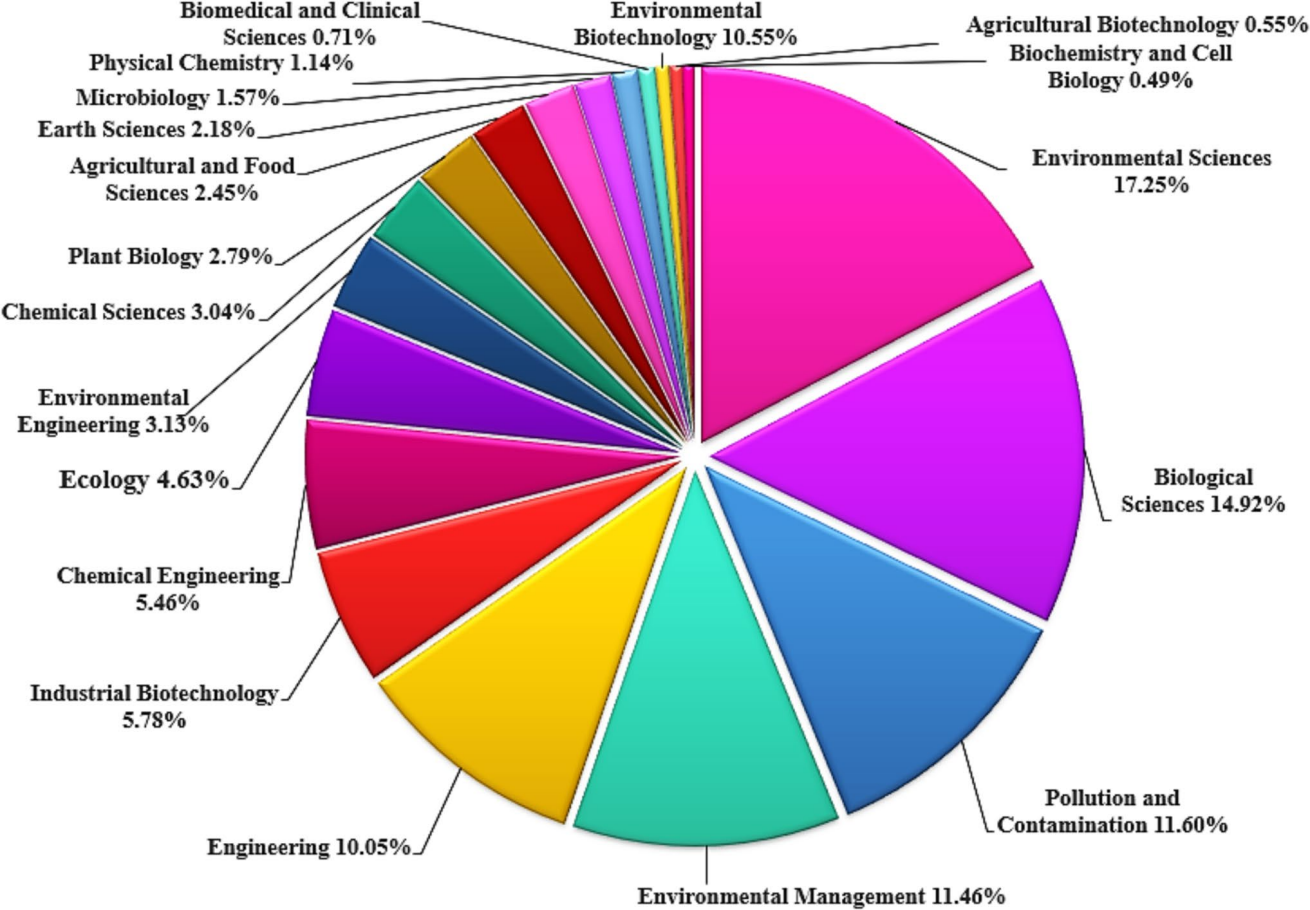
Distribution of publications by subject areas relating to microalgae-based biodegradation of SOW

Figure 5 illustrates the distribution of scientific publications based on subject areas in relation to microalgae-based biodegradation of SOW. From the results obtained, 3 main subject areas including environmental sciences, biological science, pollution and contamination contributed the most publications with 3,571, 3,090 and 2,402 records respectively, accounting for 17%, 15% and 12% of the total number of publications, respectively. This suggests that environmental sciences gained the most research attention, especially in the remediation of SOW using microalgae over the years. This distribution shows trends in research topics and that scientists are exploring different subject areas using microalgae to develop sustainable, bio-based, eco-friendly and cost-effective solutions for SOW remediation. This interdisciplinary expertise has shown microalgae-based technology for the treatment of SOW more practicable. Similar assertions were made by Radziff et al. (2021), Ubando et al. (2021), De Souza et al. (2019) and Rumin et al. (2020) after microalgae-based technology for biodegradation of organic pollutants bibliometric analyses.

### Analysis of publications in countries and collaboration networks

**Fig. 6:**
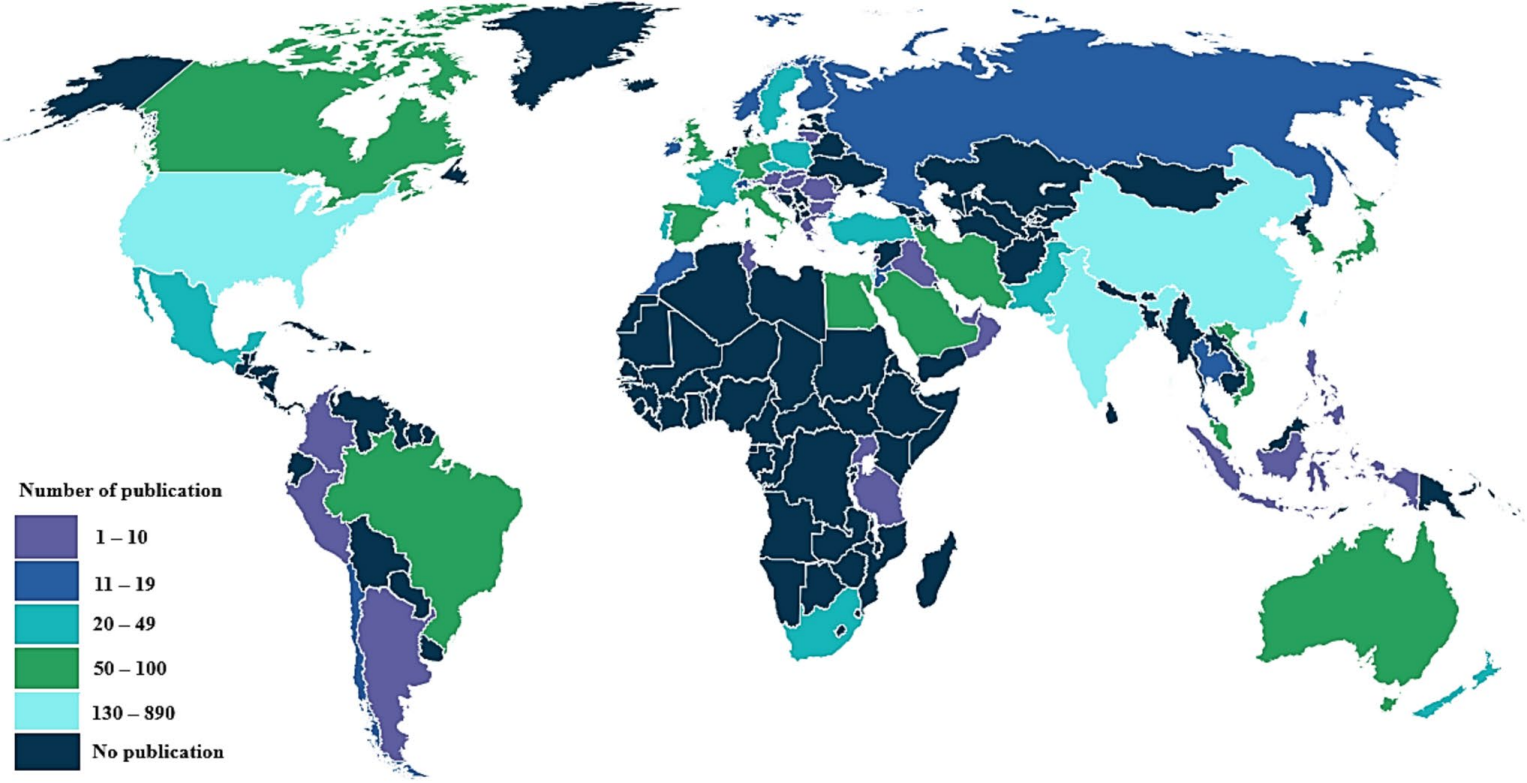
Number of the global publication across various countries in relation to microalgae-based biodegradation of SOW treatment (dimensions database)

Figure 6 represents the global publication trends across various countries and each country’s domination in relation to microalgae-based biodegradation of SOW. The data revealed that, Asia, North America and Europe regions produced the highest publication outputs. While Latin America, Africa and middle East regions produced the lowest scientific publications (Fig. 6). China, United States of America (USA), India, United Kingdom (UK), Australia, South Korea, Spain, Germany, Canada, and Egypt were the top ten productive countries publishing the most articles in relation to this research field. Among these countries, China tops the list, followed by the USA and India with 887, 354 and 213 publications, respectively, all represented by a light blue shade on the world map. The high numbers of publications observed with these countries indicates a high level of commitments in advancing research in SOW treatment towards developing a sustainable, eco-friendly and cost-effective solutions to mitigate the environmental impacts of SOW compared to other countries. Substantial increase in research publications is expected with advancements in SOW management technologies. These innovations attract scientific interest and funding, leading to more studies on sustainable SOW treatment. The noticeable low interest in this area of research in Latin America, Africa and middle East can be attributed to several factors. These include limited funding, challenges in accessing research facilities, restriction to technology, low level of collaboration, and inadequate environmental policies. Many countries in these regions tend to prioritize immediate economic development over environmental sustainability, resulting in less investment in SOW management research. There is also a significant lack of public and institutional awareness regarding the environmental and health hazards associated with SOW disposal. This coupled with a lack of technical expertise, and infrastructure for proper SOW treatment, further delays research efforts in this field. Political and economic instability, and inconsistent policies in some nations may also contribute to the lack of long-term research initiatives in this field (Miressa 2019). Strategies to address the lack of interest in SOW research in Latin America, Africa, and the Middle East is to promote awareness of its environmental and economic implications through policy advocacy and collaborations (Miressa 2019). Likewise, promoting international collaborations with institutions in regions where such research is more advanced can facilitate knowledge transfer.

**Fig. 7:**
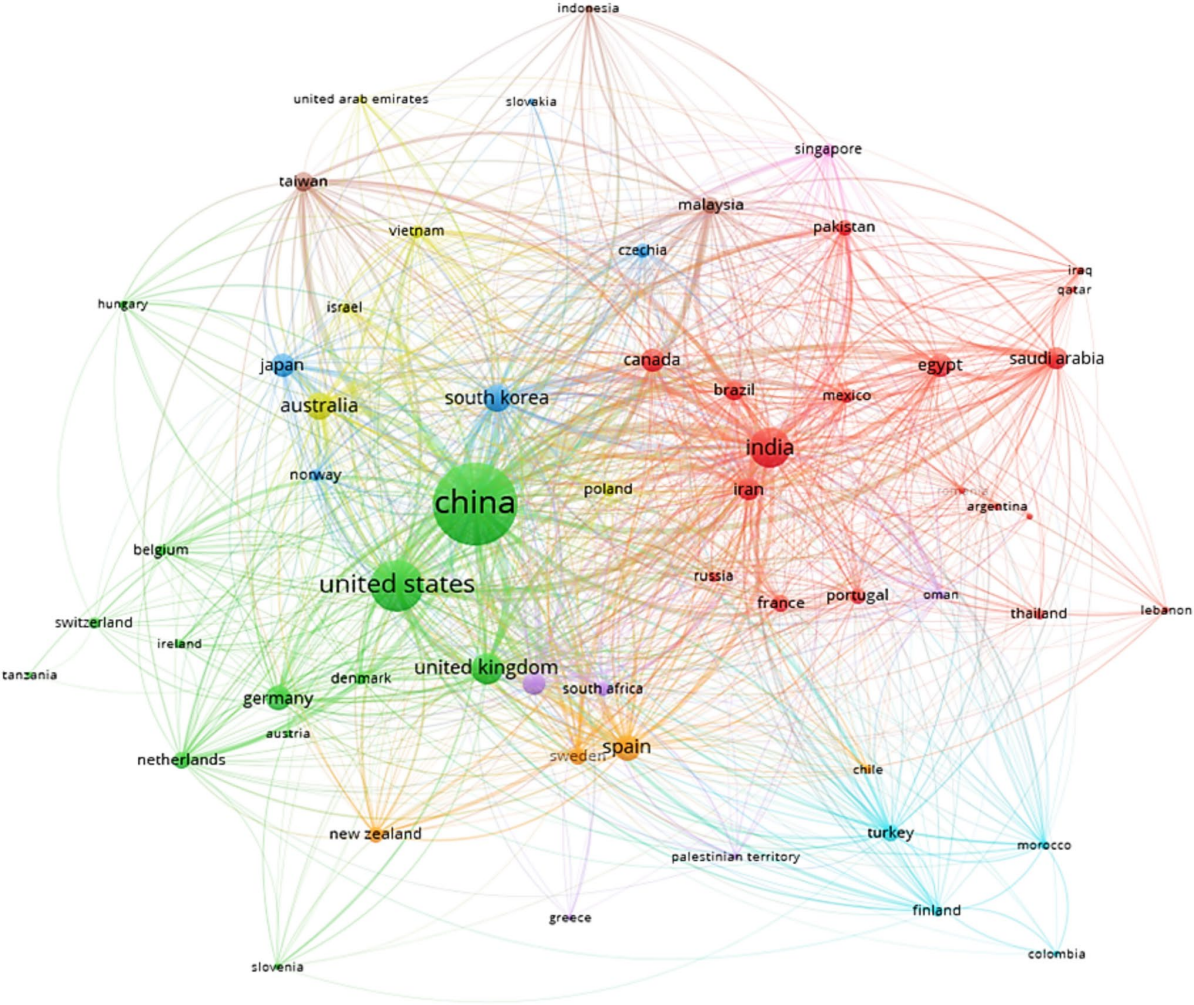
Collaboration networks among countries in relation to microalgae-based biodegradation of SOW treatment using the VOSviewer software

The collaboration networks among countries contributing to scientific publications in the field of microalgae and SOW treatment is shown in Fig. 7. A total of 9 regional clusters were observed showing collaboration among countries. The size of the circles corresponds to the volume of scientific publications produced by the respective countries, while the lines represent cooperation network among the various countries. The shorter distance between two circles indicates strong collaboration between countries. The color of the circles represents countries that belong to the same cluster. As shown in Fig. 7, the highest level of collaboration was observed among China, USA, India, UK, Spain, Australia and South Korea compared to other countries. The observed increase in publications in this study offers valuable insights for researchers to identify global trends and enhance collaboration within their respective fields of study.

The collaboration among these countries involves various activities such as joint research projects, co-authored publications, shared funding, and technology transfer agreements. For example, joint research programs could facilitate interdisciplinary studies on optimizing microalgae-based biodegradation of SOW. Also, collaborations between research institutions in countries with advanced bioremediation technology and those in regions with severe pollution challenges could result in the implementation of pilot-scale treatment systems. Academic partnerships, workshops, and international conferences will promote the exchange of knowledge on microalgae-based remediation techniques. Countries with strong funding programs, such as the USA, Germany, and China, often collaborate with developing nations by providing grants and infrastructure to support microalgae-based waste treatment research.

### Analysis of publication sources

Understanding the distribution of research articles across journals provides insights into the most influential publication sources in this field. Research articles on microalgae-based treatment of SOW have been published in 115 journals. The top 20 most productive journals in this research field are summarized in Table 2. Bioresource Technology (impact factor 9.7), Science of the Total Environment (impact factor 8.2) and Chemosphere (impact factor 8.1) were the most productive, with 249, 221, and 165 articles, receiving 22,962, 15,944, and 7,358 citations, respectively. It is also noteworthy to mention that the majority of the top 20 journals were published by Elsevier and Springer, underscoring the strong support and commitment of these publishers in this research area. This analysis highlights the key journals where researchers in this field disseminate their findings, helping to identify reputable sources for future studies and collaborations.

**Table 2:** The top 20 journals publishing microalgae research on spent oil/lubricant oil waste globally

Journal name	Total publication	Citation	Total link strength	Publisher
Bioresource Technology	249	22,962	35,194	Elsevier
The Science of The Total Environment	221	15,944	22,368	Elsevier
Chemosphere	165	7358	24,512	Elsevier
Algal Research	127	4401	1030	Elsevier
Water Research	125	9137	18,080	Elsevier
Journal of Hazardous Materials	122	9952	17,218	Elsevier
Water Science & Technology	120	2959	7645	IWA
Journal of Applied Phycology	113	4137	1056	Springer
Environmental Science and Pollution Research	109	2779	17,289	Springer
Journal of Environmental Management	79	4396	12,148	Elsevier
Environmental Technology	55	1228	6797	Taylor and Francis
Environmental Pollution	54	3121	7631	Elsevier
Biodegradation	49	402	1150	Springer
Environmental Science and Technology	41	3540	3812	Springer
Ecotoxicology and Environmental Safety	37	2874	4416	Elsevier
Scientific Reports	36	922	4903	Nature
Water Environment Research	30	540	5061	Wiley
Journal of Environmental Sciences	24	849	3461	Elsevier
International Journal of Phytoremediation	22	415	4409	Taylor and Francis
Plos One	20	597	1218	PLOS

### Analysis of productive authors

The top authors publishing scientific articles in relation to SOW or used lubricant oil waste were identified and analyzed based on bibliometric indicators. These indicators including author names, identity number, current affiliations, total citations, number of publications, h-index, and country of origin. The top 20 authors in this field of research, based on publication output, are presented in Table 3. Of the 10,038 authors identified from the employed database, the top three researchers who actively published articles relating to the study of SOW or used oil waste degradation treatment were Ruan R. (USA), Chen P. (USA) and Gong DF. (China). Among these authors, Ruan, R., from the University of Minnesota Twin City, USA was the most productive author, with 39 publication and 4649 citations compared to other authors. Similarly, the h-index of the author Ruan R., (103) was higher compared to h-index obtained for rest of the authors. The h-index has been used to assess researchers’ overall impact, as it reflects both the quantity of publications and number of citations (Tzitzikas and Dovas 2024; Srinivas and Bharathi 2024). More active authors were linked with institutions in USA and China known for their strong commitment to environmental protection and searching for sustainable, eco-friendly strategies to mitigate the harmful effects of SOW pollutants. The number of publications by the top 20 authors were different in their approach and findings, indicating that all listed authors are playing crucial roles in advancing the research area of microalgae-based biodegradation treatment of SOW.

**Table 3:** The top 20 authors publishing microalgae research on SOW globally

Authors names	Identity number	Total publication	Total citation	Total link strength	h-index	Current affiliation	Country
Ruan, Roger	6763	39	4949	14,876	103	University of Minnesota Twin City	USA
Chen, Paul	1284	28	4501	13,572	82	University of Minnesota Twin City	USA
Gong Duan Fan	2161	27	905	6068	51	Fuzhou University	China
Liang, Heng	4589	25	947	9980	75	Harbin Institute of Technology	China
Zhou, Wenguang		23	2886	10,510	45	Nanchang University	China
Ponnusamy Senthil Kumar	4096	28	1,118	5048	46	Centre for Pollution Control & Environmental Engineering, Pondicherry University	India
Jiu-Hui Qu	6450	17	830	3095	53	Research Center for Eco-Environmental Sciences	China
Mostafa, ElSheekh		15	614	701	49	Tanta University	Egypt
Lu, Qian	4927	14	1237	8854	25	Jiangsu University of Science and Technology	China
Min, Min	5416	12	3200	6838	64	University of Minnesota Twin City Twin City	USA
Pan, Gang	6011	12	569	1261	64	York St John University	UK
Zhao, Yongjun		11	619	5327	82	Hangzhou Normal University	China
Chang, Jo-Shu	1172	11	716	4224	99	Tunghai University and National Cheng Kung University	Taiwan
Lei, Zhongfang	4308	11	896	5048	55	University of Tsukuba	Japan
Barceló, Damià	569	11	2359	3748	100	Institute of Environmental Assessment and Water Research	Spain
Ho, Shih-Hsin	3132	10	830	3010	86	Harbin Institute of Technology	China
Liang Wang	8341	10	598	5445	80	Shanghai University	China
Lei Li	4437	10	660	7016	56	State Key Laboratory of Pollution Control and Resource Reuse	China
Liu, Yuhuan	4850	10	1719	5080	46	Nanchang University	China
Finlay, John A	2316	9	511	2032	62	Newcastle University	UK

**Fig. 8:**
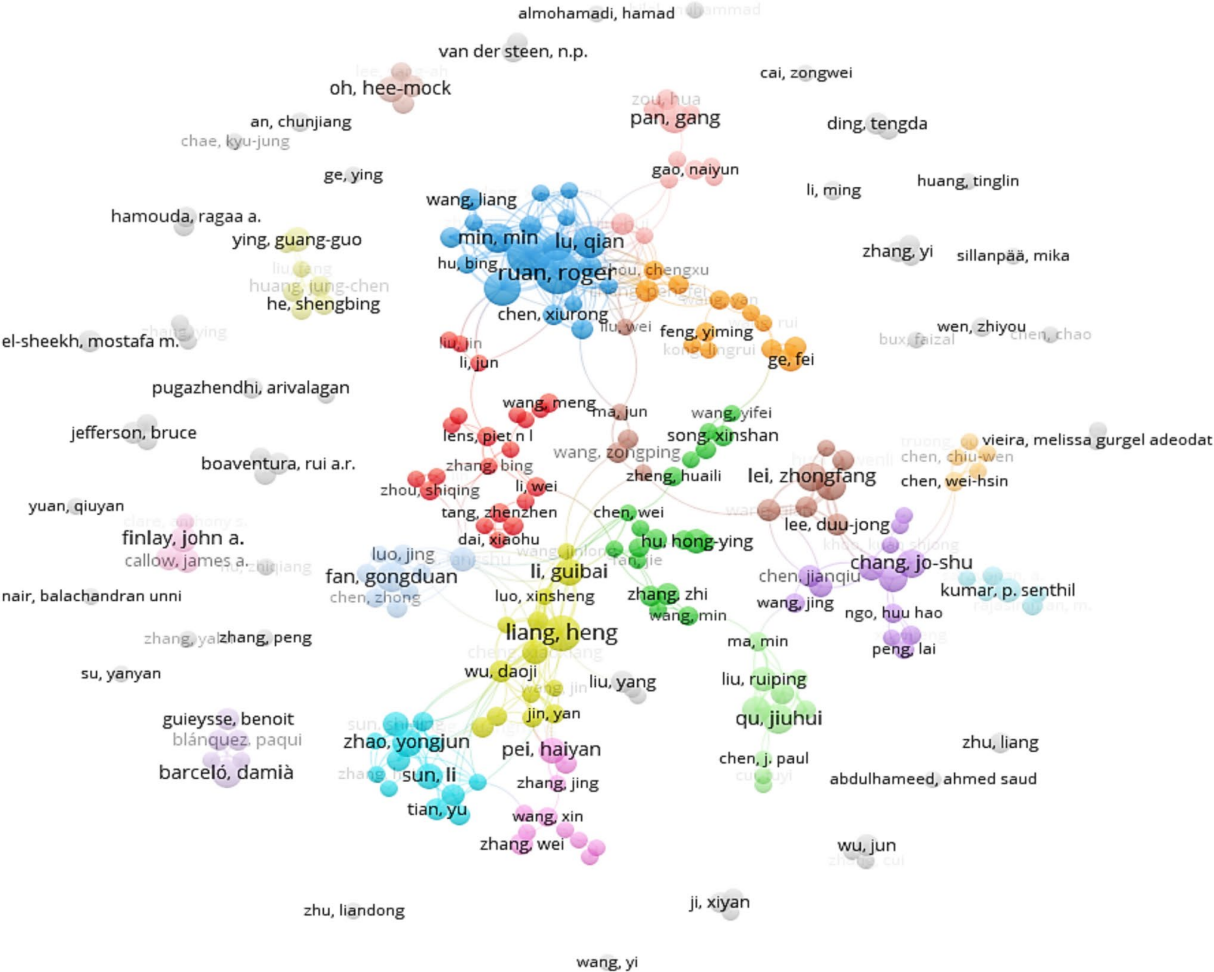
Co-authorship analysis of 251 researchers in relation to microalgae-based biodegradation of SOW treatment using the VOSviewer software

**Fig. 9:**
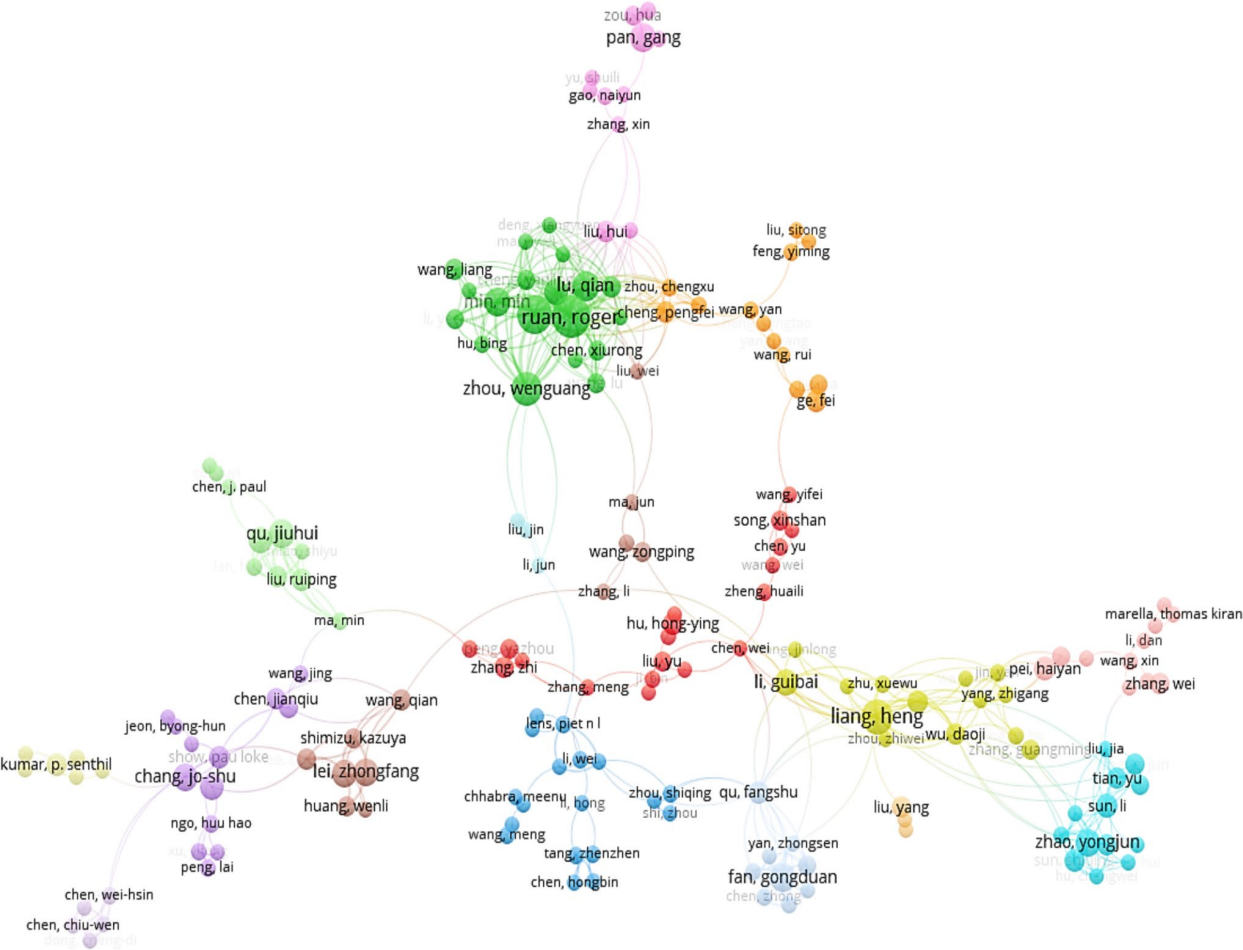
Co-authorship analysis of 179 researcher in relation to microalgae-based biodegradation of SOW treatment using the VOSviewer software

The co-authorship analysis is illustrated in Figs. 8 and 9. In Fig. 8, a total of 251 researchers were identified with co-authorship links of 552, total link strength of 1493 and 54 clusters. As shown in Fig. 8, some of the authors among the 251 identified were not connected or cooperating with each other, which led to no contact with the entire collaboration network. For each of the 251 authors, the total strength of the co-authorship links with other authors were calculated. The authors with the greatest total link strength were selected (Fig. 9). In Fig. 9, a total of 179 authors, co-authorship links of 516, total link strength of 1288 and 16 clusters were observed showing the relatedness of researchers based on the co-authored publications. The size of the circles corresponds to the number of co-authored publications produced by the respective researchers, while the lines indicate cooperation network among the co-authors. The shorter distance between the circles suggests strong co-authorship between authors. The color of the circles shows co-authors that belong to the same cluster. The highest level of collaboration was observed among the Ruan R. (USA), Chen P. (USA), and Gong DF. (China), and Liang H. (Chian) the green color clusters.

### Analysis of productive organizations

From 1882 to 2023, 339 organizations were involved in the research of microalgae and SOW treatment. The organizations included universities, research institutions, research centers and laboratories. Table 4 shows the top 20 productive organization globally publishing article related to microalgae and SOW treatment. The results of the analysis indicate that sixteen of the organizations were from China and one from USA, India, Taiwan and Japan. The concentration of the research organization in China a developing country compared to the established organization in developed countries such as the USA, Taiwan and Japan suggests a significant shift in the global microalgae research. This trend could indicate China’s increasing investment in microalgae research and SOW treatment, leading to the rise in scientific publications from this country (Qi et al. 2019). Interestingly, the results obtained from this study also contradict the common belief that developed countries are the main leaders in research productivity in SOW biodegradation (Börner et al. 2018).

**Table 4:** The top 20 productive organizations globally during the period of 1882 – 2023

Organization	Identity No	Publication	Citation	Total link strength	Country
Harbin Institute of Technology	778	72	3441	25,008	China
University of Chinese Academy of Sciences	2136	61	2871	21,359	China
University of Minnesota Twin City	2248	40	5429	15,515	USA
Research Center for Eco-Environmental Sciences	1664	36	2076	11,862	China
Chongqing University	338	33	1059	10,351	China
Tongji University	1986	29	854	6975	China
Tsinghua University	1995	28	3302	6837	China
Shandong University	1767	27	658	9353	China
Anna University	75	22	1533	7732	India
Hohai University	822	22	671	7626	China
King Saud University	1136	22	1031	6879	China
Nanchang University	1366	21	2555	9870	China
National Cheng Kung University	1401	20	1451	8748	Taiwan
Shanghai Jiao Tong University	1780	20	912	4565	China
State Key Laboratory of Pollution Control and Resource Reuse	1880	19	553	5197	China
University of Tsukuba	2357	18	1267	6236	Japan
Jiangnan University	1046	17	490	3884	China
Fudan University	682	17	885	5734	China
Chinese Academy of Sciences	330	16	491	4523	China
Fuzhou University	694	16	898	4639	China

In terms of the research productivity, Harbin Institute of Technology and University of Chinese Academy of Sciences in China were the most productive with 72 and 61 publications with 3441 and 2871 citations respectively, followed by University of Minnesota Twin City in the USA with 40 publications and 5429 citations. These findings further align with the results obtained from the bibliometric analysis of countries and authors. Other research organization in China, Anna University in India, National Cheng Kung University in Taiwan, and University of Tsukuba in Japan, also contributed to several publications in this field of research.

### Analysis of documents citation

The number of top citations per document or articles related to microalgae and SOW treatments are listed in Table 5. A total of 2500 articles were observed, and the number of citations were significant different, indicating that all listed articles have demonstrated a significant impact in advancing the field of microalgae and SOW treatments. The most cited article was Wang (2008) with the id number 907 and has received more than 2211 citations compared to the other documents. This implies the major impact and recognition of the article globally, showing its relevance and importance within this research field. Four other articles including Davis (2003), Vijayaraghavan (2008), Pittman (2010) and Sheng (2004) also exhibited high citation number of 1978, 1516, 1251 and 947, respectively, further demonstrating their substantial impacts and recognitions within this subject area and the research community.

**Table 5:** Documents and the number of citations related to the keyword search 14 (Table 2)

Documents	Identity number	Citation	Total link strength
Wang et al. (2008)	907	2211	904
Davis et al. (2003)	762	1978	208
Vijayaraghavan et al. (2008)	2385	1516	754
Pittman et al. (2010)	147	1251	971
Sheng et al. (2004)	1800	947	116
Mehta et al. (2005)	570	694	924
Igiri et al. (2018)	434	678	249
Xu et al. (2020)	2268	656	119
Li et al. (2011)	438	622	316
Wang et al. (2009)	577	588	151
He et al. (2014)	548	573	552
Zhu et al. (2013)	480	434	444
Lim et al. (2010)	135	428	139

## Common constituents of spent oil wastes and degradation

### Alkanes, aromatics, polychlorinated biphenyls and phenolic compounds

Alkanes, monoaromatics, PAHs, PCBs, and phenol compounds are major constituents of SOW (Kaleem et al. 2023; Satpati et al. 2023). They are characterized as a persistent hydrocarbon in the environment due to their low water solubility, low volatility, and resistance to biodegradation (Chauhan et al. 2024; Hoque et al. 2023; Wang et al. 2023). These organic compounds cause ecological problem when discharged into the environment. They have been described to cause harmful effects on aquatic life, plant and animals, thus, the need for their removal. Many studies on the chemical, physical, and biological treatments have been conducted to remediate these hazardous SOW contaminants (Kaleem et al. 2022; Touliabah et al. 2022). Among these treatments, microbial degradation was found to be the most environmentally friendly (Kaleem et al. 2023; Touliabah et al. 2022). Many microorganisms with the capability of degrading SOW pollutants have also been reported and most biodegradation studies have focused primarily on the use of bacteria and fungi (Chauhan et al. 2024; Hoque et al. 2023; Wang et al. 2023). Lately, there have been studies showing the capability of microalgae. Tables 6 and 7 show degradation of alkane and aromatic

**Table 6:** Degradation of alkane compounds by microalgae

Spent oil waste pollutants	Degradation rate (%)	Time (days)	Microalgae	References
Alkanes				
Decane	75.32	30	*Anabaena oryzae*	(Hamouda et al. 2016)
Undecane	80.25	30	*A. oryzae*	(Hamouda et al. 2016)
Hexane	100	7	*S. vacuolatus*	(Eregie et al. 2023)
Dodecane	96.50	7	*Chlamydomonas* sp	(Ichor et al. 2016)
Tridecane	70.2	7	*Nitzschia linearis*	(Hammed et al. 2016)
Hexadecane	45	3	*Phormidium* sp	(Dell’Anno et al. 2021)
Heptadecane	90.62	30	*C. kessleri*	(Hamouda et al. 2016)
Octadecane	89.53	30	*C. kessleri*	(Hamouda et al. 2016)
Nonadecane	95	7	*Prototheca* sp	(Dell’Anno et al. 2021)
Tetracosane	79.5	5	*Phaeodactylum tricornutum*	(Pi et al. 2015)
Pentacosane	80	5	*Dicrateria* sp	(Pi et al. 2015)

compounds by microalgae.

**Table 7:** Degradation of Spent oil waste aromatic compounds by microalgae

Pollutants	Degradation rate (%)	Days	Microalgae	References
Monoaromatic compounds				
Benzene	97	7	*Thalassiosira sp*	(Li and Meng 2023)
Toluene	63	2	*Parachlorella kessleri*	(Takáčová et al. 2015)
Ethylbenzene	62	2	*P. kessleri*	(Takáčová et al. 2015)
Xylenes	40	2	*P. kessleri*	(Takáčová et al. 2015)
Benzo(a)pyrene	95	3	*Selenastrum capricornutum*	(García de Llasera et al. 2022)
Benzo(a)pyrene	99	0.63	*Scenedesmus acutus*	(García de Llasera et al. 2022)
Polycyclic aromatic compounds				
Phenanthrene	96 and 98	4 and 30	*S. capricornutum* *G. pectoral*	(Subashchandrabose et al. 2013) (Hoque et al. 2023)
Fluoranthene	100	4	*S. capricornutum*	(Subashchandrabose et al. 2013)
Naphthalene	90	2	*Oscillatoria sp. Phormidium tenue*	(Żyszka-Haberecht et al. 2019)
Anthracene	92.28 and 98	3 and 30	*Eustigmatos viridis* *G. pectoral*	(Żyszka-Haberecht et al. 2019) (Hoque et al. 2023)
Pyrene	33	7	*Anabaena fertilissima*	(Patel et al. 2020)
Polychlorinated biphenyls (PCBs)				
Polychlorinated biphenyls	84.4	25	*Anabaena strain PD-1*	(Kaleem et al. 2023)
Phenolic compounds				
Catechol and hydroxytyrosol,	70		*Ankistrodesmus braunii* *S. quadricauda*	(Touliabah et al. 2022)

### Impacts of spent oil wastes (SOW)

#### Environmental impact spent oil wastes

**Fig. 10:**
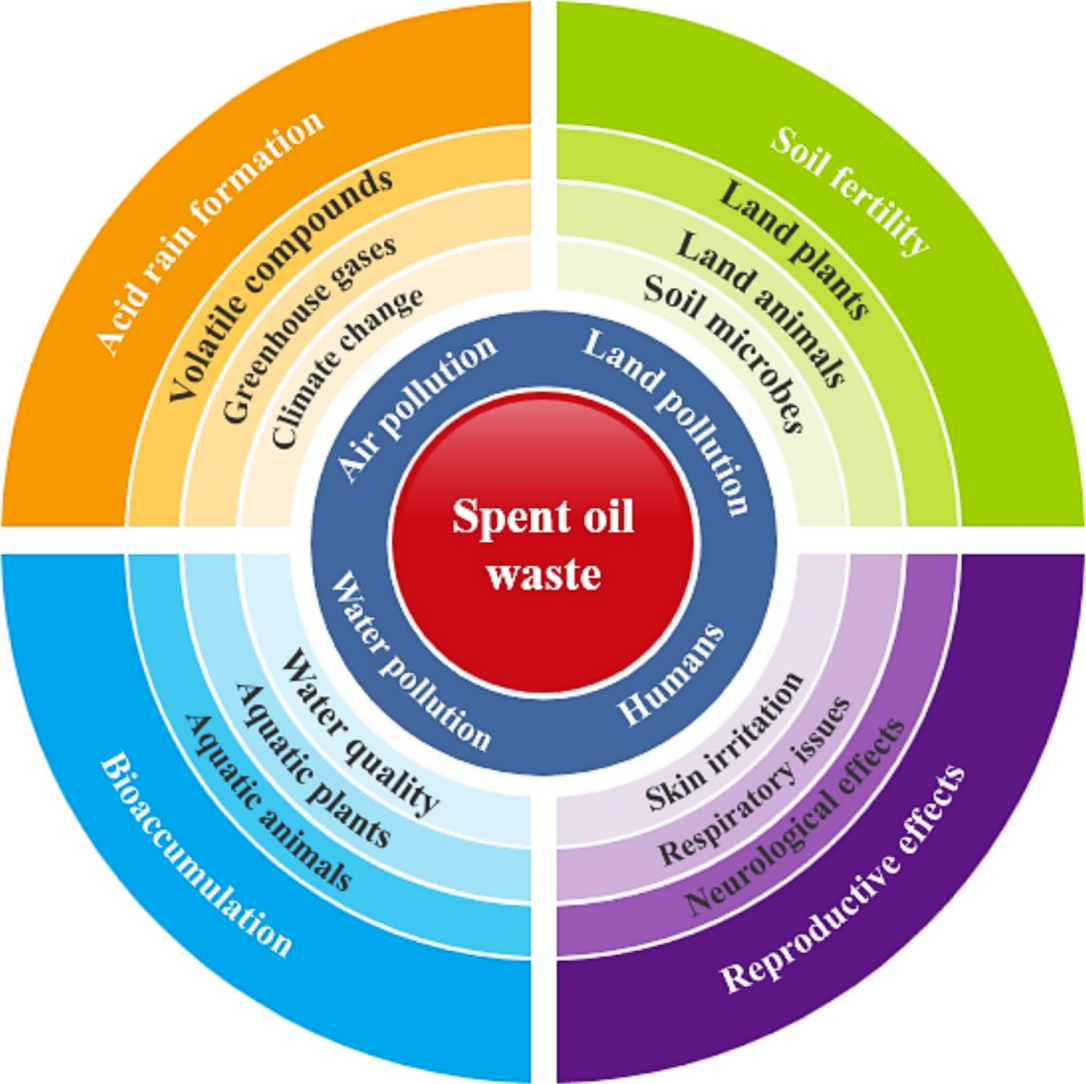
Spent oil waste impact on the environment and human health

There is a lot of reports about the severe damage caused by SOW spills on the ecosystems (Dey et al. 2024; Shah and Soni 2024; Kanungo et al. 2024). Soil pollution (Fig. 10) is one of the main problems caused by the improper disposal of spent oil wastes. SOW wastes dumped in landfills sites have hazardous compounds that could leaked into the soil. These hazardous compounds could be bioaccumulated over time. Soil pollution does not only affect plant growth, but it is also unhealthy for humans and causes death in animals who consume plants growing in the polluted area or landfills (Dey et al. 2024). Uquetan et al. (2017) reported that the presence of SOW in the soil adversely affected the physical, chemical, and microbiological properties of the soil (Olukunle and Sanusi 2015), these in-turns affected the germination of crop seeds as well as the growth of cultivated crops. Ohanmu et al. (2019) also reported the impact of lubricant oil waste accidents on vegetation and land. Their report shows that after forty years of several clean-ups, the soils are still contaminated with lubricant oil waste. The negative effects like brownish leafless vegetation and barren lands were also reported to be persistent after 40 years.

Air pollution occurs when SOW is incinerated at landfills sites, emits gaseous compounds that contaminate the atmosphere (Fig. 10) and destroys our ozone layer. Sulphur dioxide, carbon monoxide, dioxins, methane gas, carbon dioxide, nitrogen oxides, particulate matter, and volatile organic compounds produced during the burning of used oil wastes are toxic to humans with potential to cause health problems such as respiratory diseases when inhaled. The volatile organic compounds and particulate matter produced during incineration causes the irritation of the lungs, asthma, and bronchitis (Ohanmu et al. 2019). These greenhouse gases are the main source of global warming, facilitating global temperature rise by 1.4 degrees Fahrenheit (Dey et al. 2024). Potential harmful effect of greenhouse gasses includes extreme weather changes such as heavy rainfall, strong storms, tornadoes, hurricanes, whirlwinds, windstorms, and global extreme heat (Dey et al. 2024).

Water pollution results from improper disposal of SOW directly or indirectly to water bodies. This oil waste can also leach into groundwater during activities such as dust cure and road oiling. Hazardous hydrocarbon compounds from SOW can also leach into streams and other water bodies. Illegal disposal or deposition of SOW in water bodies can cause the loss and toxification of aquatic creatures and indirectly harmful to humans who consume contaminated fishes (Fig. 10). For instance, Shah and Soni (2024) reported the Exxon Valdez accidental spent oil land spill, their result revealed that even many years after the disaster, significant genotoxic damage still exists in mussels and oysters obtained from the polluted land. The authors also reported that one litre of SOW could deplete the oxygen of a million litres of water, causing the suffocation of aquatic creatures and preventing plants from photosynthesizing.

#### Health impact spent oil wastes

Improper disposal of SOW has been linked to be the causes of many diagnosed diseases (Ohanmu et al. 2019). The health problems associated with SOW may be through any or combinations of the following routes: contaminated food, water, and emission, from landfills and open dumpsites (Fig. 10) (Ohanmu et al. 2019). Allergies, skin irritations, gastrointestinal issues psychological disorders, respiratory problems, birth defects and death are the major human health problems associated with improper disposal of SOW (Ohanmu et al. 2019). Polycyclic aromatics compounds are the most hazardous components of SOW with chronic and carcinogenic effects due to their recalcitrant nature (Romero et al. 2018). Alrumman et al. (2015) reported the chronic effects of naphthalene, a polycyclic aromatic hydrocarbon when consumed include hepatitis, kidneys failure, heart rate malfunction, lungs disease, destruction of intestinal organs and nervous system disorder. Also, consumption of SOW contaminated food reduces the ability of animals to absorbs and break down food (Romero et al. 2018).

#### Factors affecting spent oil waste hydrocarbon biodegradation

**Fig. 11:**
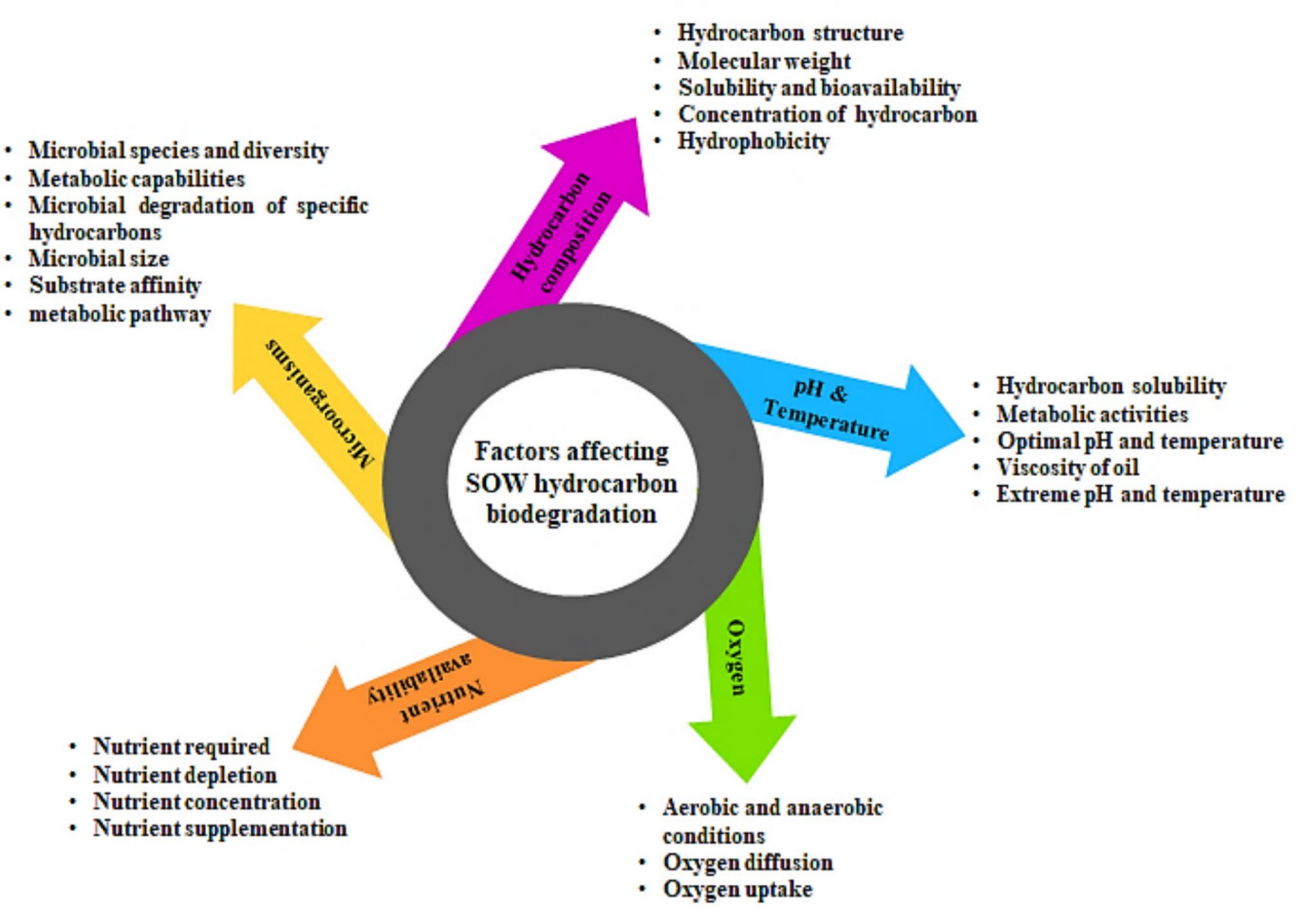
The factors influencing SOW hydrocarbon biodegradation

SOW are complex mixtures of hydrocarbons contaminants that degrade at different rates depending on their chemical structure and concentration (Koh and Khor 2022). The factors influencing waste lubricant hydrocarbon biodegradation are shown in Fig. 11. Typically, the biodegradation rate increases with decreasing molecular weight and complexity of the chemical structure of the hydrocarbon (Touliabah et al. 2022). The saturated compounds are the n-alkanes with a carbon length ranging from C10–C25, and those with shorter chain length (C10–C16) are more easily degraded by microorganisms due to their water solubility and bioavailability. Long chain n-alkanes (C25–C40) are hydrophobic solids and are more resistant to degradation. Branched-chain alkanes and cycloalkanes are also degraded slowly compared to their corresponding straight alkanes. Aromatic compounds are recalcitrant hydrocarbons that have great resistance to degradation (Touliabah et al. 2022). Previous studies have shown that the rate of microbial uptake and biodegradation of aromatic hydrocarbons is dependent on the solvent solubility of the hydrocarbons (Eregie and Jamal-Ally 2019; Koh and Khor 2022; Touliabah et al. 2022). Usually, contaminant concentration greater than 10% v/v leads to a decrease in microbial activity and biodegradation rate (Li and Meng 2023). The concentration of contaminants directly affects microbial activity (Koh and Khor 2022).

Temperature plays very important roles in the biodegradation of hydrocarbon (HC) pollutants by its direct effect on the chemistry and physiology of the HC and microbial activities (Fig. 11) (Kumar and Shukla 2023). Temperature influences the solubility of hydrocarbons, with higher temperatures leading to increased HC solubility which ultimately increases the bioavailability of hydrocarbon molecule. Oxygen solubility decreases with increasing temperature, which reduces the metabolic activity of aerobic microorganisms (Koh and Khor 2022). Biodegradation of hydrocarbon occurs over a broad temperature range. Most studies tend to focus on mesophilic temperatures rather than the efficiency of transformations at very low or high temperatures (Koh and Khor 2022). The viscosity and solubility of lubricant oil increases with low temperature, therefore, causes the lubricant oil to become toxic and recalcitrant to microbial attack (Koh and Khor 2022). Some hydrocarbons are more soluble at lower temperatures and some low-molecular-weight aromatics are more soluble at a higher temperature (Li and Meng 2023). Although hydrocarbon biodegradation occurs over a wide range of temperatures, the rate of biodegradation generally decreases with decreasing temperature and increases with increasing temperature (15 to 40 °C) (Koh and Khor 2022). This is because enzyme activity increases with temperature, facilitating the required biochemical reactions essential for the biodegradation process to occur.

Aerobic conditions are generally considered necessary for extensive degradation of oil contaminants in the environment since major degradative pathways for both alkanes and aromatics involve oxygen (Li and Meng 2023; Kumar and Shukla 2023). Oxygen availability is dependent on the ability of oxygen to move or diffuse through the contaminants as well as on the uptake rate by microorganisms. The primary rate-limiting factor in aerobic biodegradation is the delivery of oxygen. Previous studies have shown that the addition of oxygen increases degradation rates, and oxygen depletion leads to a reduction in biodegradation activities (Li and Meng 2023; Kumar and Shukla 2023). Touliabah et al. (2022) reported that under the aerobic condition, benzene, toluene, ethylbenzene, and xylenes (BTEX) compounds degraded faster compared to the anaerobic environment. Anaerobic hydrocarbon degradation has been shown in some studies to occur only at negligible rates. Recent studies have shown that anaerobic hydrocarbon metabolism may be an important process in certain conditions (Li and Meng 2023; Kumar and Shukla 2023). The biodegradation of BTEX compounds has been demonstrated to occur under a variety of anaerobic conditions (Touliabah et al. 2022). Studies have also demonstrated that in some marine sediment, PAHs and alkanes can be degraded under sulphate-reducing conditions at similar rates to those under aerobic conditions (Touliabah et al. 2022; Pandey et al. 2016).

Microorganisms need nutrients for growth and require mineral nutrients such as carbon, nitrogen, phosphorus, potassium, sulphur, and magnesium for metabolic processes (Fouad et al. 2022). In contaminated sites, where the carbon levels are often high due to the nature of the pollutant, available nutrients could become rapidly depleted during microbial metabolism (Fouad et al. 2022). Many studies have shown that the types and quantities of nutrients present in the system play a much more important role in limiting the rate of hydrocarbon degradation (Fouad et al. 2022; Touliabah et al. 2022). Higher concentration of nutrients was reported to cause the toxicity of the growth medium and exert toxic stress on degrading microorganisms making them unable to carry out their metabolic activities. Whilst lower nutrient concentration leads to competition among microorganisms due to insufficient nutrient source (Fouad et al. 2022). Nutrients especially, nitrogen, phosphorus, and in some cases, iron are very important ingredients for successful biodegradation of hydrocarbon pollutants. Some of these nutrients in excess or limited amounts could become limiting factors, affecting the biodegradation process (Fouad et al. 2022). Nutrient supplementation can be used as a tool to increase biodegradation rates (Fouad et al. 2022). The ability of a microorganism to grow in any system depends on the organism’s ability to utilize available nutrients. The amount of nutrients required to degrade a certain amount of oil is not fully understood (Fouad et al. 2022). Further work in this regard would benefit future bioremediation trials.

Like other environmental parameters, pH also plays a vital role in determining the ability of microorganisms to grow and degrade hydrocarbon in the environments (Fig. 11) (Bacosa et al. 2022). Most commonly, microorganisms grow optimally within a narrow pH range between 6.7 and 7.5 during hydrocarbon degradation. The metabolic activities of microorganisms in a system can often be directly related to the pH (acidity or alkalinity) of the system under study. Studies have indicated that microorganisms naturally change the pH of their environment via the production of by-products that are either acidic or basic (Bacosa et al. 2022). The production of organic acid metabolites tends to lower the pH of the microbial environment, whilst the release of basic organic metabolites increases the pH of the microbial environment. Higher rates of biodegradation are observed at neutral pH conditions. Extreme pH, as can be observed in some environments, and were reported to affect microorganism’s capability to degrade hydrocarbons (Bacosa et al. 2022).

The composition of microorganisms in an environment plays a crucial role in determining the rate and efficiency of biodegradation (Fig. 11). This is attributed to the diverse metabolic capabilities exhibited by different microbial species (Koh and Khor 2022). The metabolic proficiency of microorganisms varies based on the nature of pollutants present. A specific microorganism might be more effective in degrading one type of HC while proving ineffective against another. Due to this variability, a pivotal aspect of any bioremediation study involves isolating and identifying microbes capable of degrading specific contaminants. The size of the microbial population is another factor impacting the biodegradation rate. Studies have demonstrated that an increase in microbial population correlates with a higher rate of hydrocarbon degradation. This suggests that the biodegradation rate is proportionate to the size of the microbial population (Koh and Khor 2022).

#### Degradation mechanisms used by microalgae for biodegradation of organic hydrocarbons

##### Alkane catabolic mechanism

**Fig. 12:**
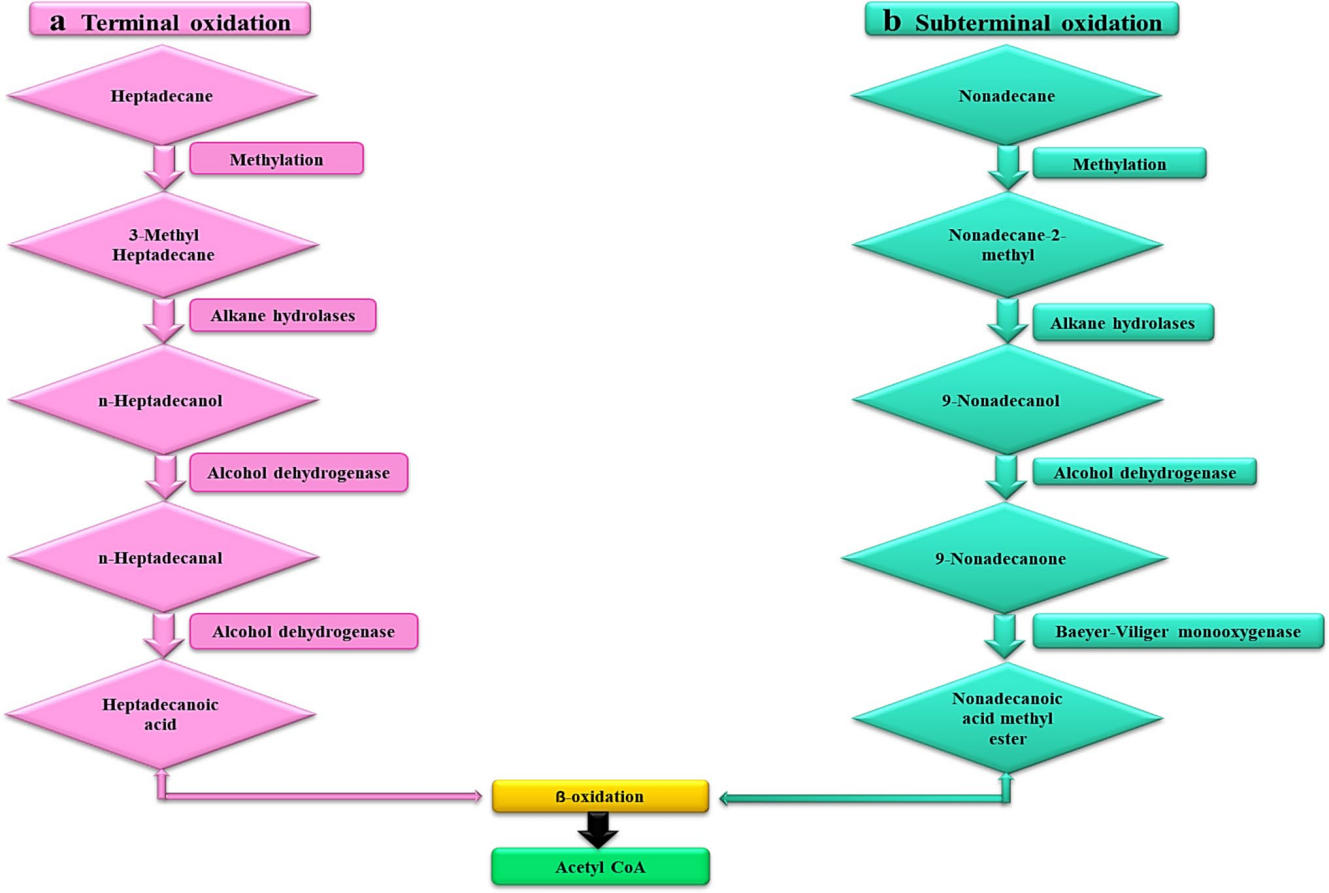
The proposed pathway showing enzymatic degradation of alkanes (heptadecane and nonadecane) by microalgae. **a** Terminal oxidation pathway; **b** Subterminal oxidation

Alkanes are organic pollutants that are prevalent in nature (Mahmoud 2023) and are listed as the dominant constituents of SOW. Studies have shown that microalgae species such as *Chlorella*, *Chlamydomonas*, *Spirulina*, *Scenedesmus*, *Nostoc*, *Oscillatoria*, *Desmodesmus*, and *Ulva lactuca* could degrade SOW contaminants (El-Sheekh et al. 2013; Hammed et al. 2016; He et al. 2016). For alkanes, the aerobic degradation is divided into two oxidation mode and these modes are known as terminal oxidation and subterminal oxidation (Eregie et al. 2023; Dell’ Anno et al. 2021). These oxidation modes take place at different carbon positions and form various end products (Wang et al. 2023). The oxidation of alkanes by microalgae is primarily carried out by specific enzymes such as alkane hydroxylases, alkane monooxygenases, alcohol and aldehyde dehydrogenases (Dell’Anno et al. 2021). These enzymes play a crucial role in the breakdown of alkane structures into simpler and less toxic compounds by increasing the hydrophilicity of the alkanes (Wang et al. 2023; Dell’Anno et al. 2021). For example, for the terminal oxidation (Fig. 12), the alkane degradation usually starts with the oxidation of a methyl group hydrocarbon via enzymes called alkane hydroxylases to form an alcohol. The alcohol formed is subsequently oxidized by alcohol dehydrogenase to form the corresponding aldehyde. The aldehyde is further metabolized via the enzyme aldehyde dehydrogenase to produce a fatty acid (Wang et al. 2023). The fatty acids are further oxidized via baeyer–villiger monooxygenase system to acetyl-CoA and to carbon dioxide and water (Fig. 12) (Wang et al. 2023). In another route, which is the subterminal oxidation, the alkane methyl group is broken down into secondary alcohol, which is converted into a corresponding ketone, then oxidized via baeyer–villiger monooxygenase to an ester. The ester is further hydrolyzed by an esterase enzyme to form a fatty acid ester (Fig. 12) (Olajire and Essien 2014). These enzymatic processes, coupled with the downstream metabolic pathways like the β-oxidation of fatty acids and the tricarboxylic acid (TCA) cycle, enable microalgae to utilize HCs as carbon and energy sources for growth.

##### Aromatic hydrocarbon catabolic mechanism

**Fig. 13:**
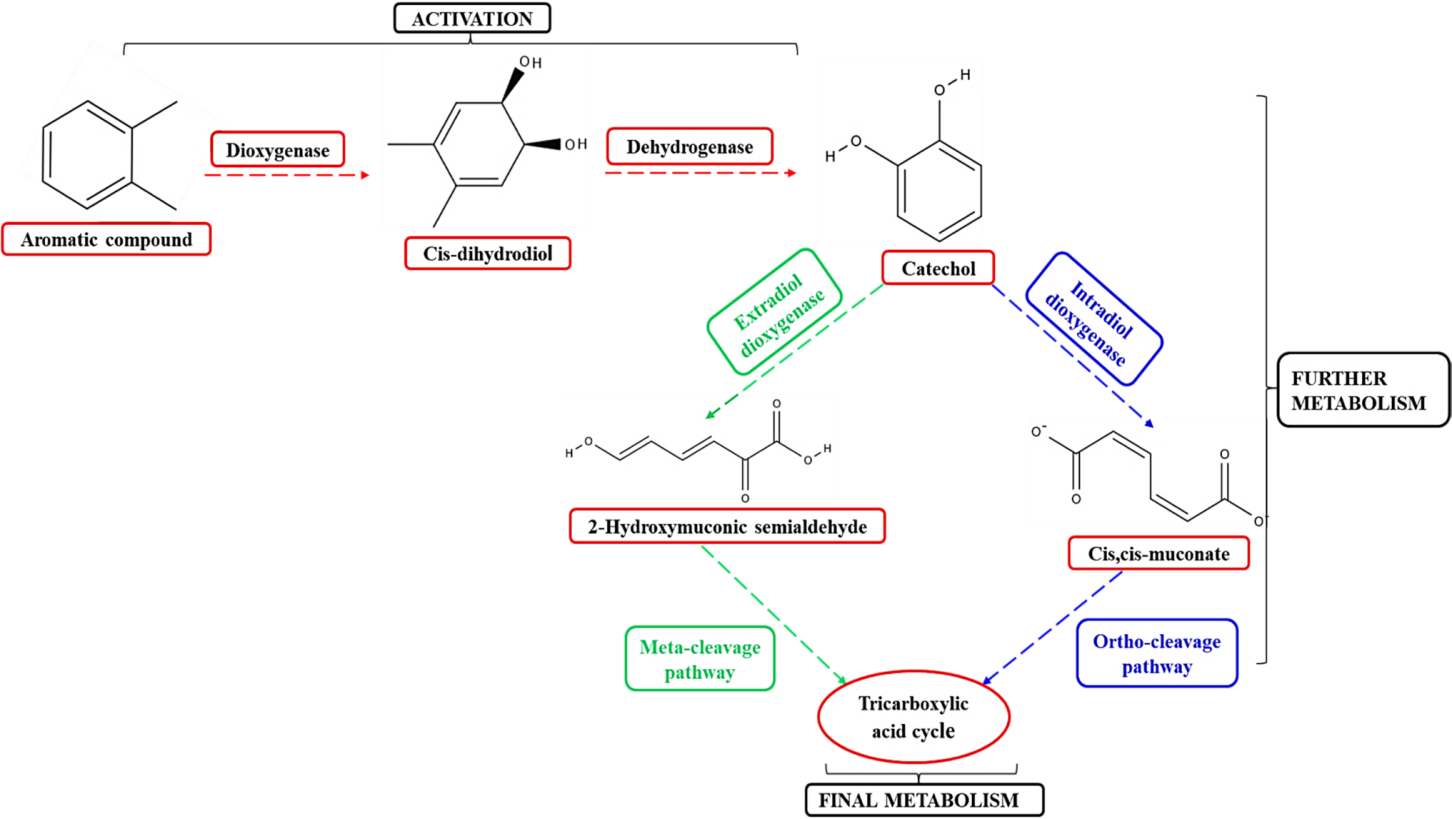
The proposed pathway showing the enzymatic degradation of aromatic compound by microalgae

Aromatic HCs are the major compounds of SOW. Due to the persistence of these compounds in the environment, the oxidation of aromatic HCs is more difficult compared to alkanes (Nagy et al. 2024). Different microalgae have been reported to biodegrade aromatic HCs, in which biodegradation of naphthalene, benzo(a)pyrene, pyrene, phenanthrene, anthracene and fluorene have been the most researched. To date, not much is known about the metabolic pathway of the aromatic HCs because the enzymatic pathways vary among microalgal species (Patel et al. 2020; Tomar and Jajoo 2021; Othman et al. 2023). Aromatic degrading enzymes are crucial in the breakdown of aromatic HCs because they initiate the enzymatic degradation of aromatic rings enabling the microalgae to use the HCs as a carbon source (Nagy et al. 2024). Aromatic degrading enzymes, such as monooxygenases, dioxygenases, dehydrogenase, superoxide dismutase, catalase, and peroxidase have been detected in many microalgae species (Nagy et al. 2024). For example, the aerobic catabolism of aromatic HCs is an oxygenase-mediated process, which involves two major steps including the activation of the ring and ring cleavage (Fig. 13) (Patel et al. 2020). Enzymes such as monooxygenases and dioxygenases initiate the breakdown of aromatic HCs by oxidizing and cleaving the aromatic ring via hydroxylation to form a cis-dihydrodiol (Fig. 13) (Imam et al. 2022; Hoque et al. 2023; Nagy et al. 2024). For further metabolism, this cis-dihydrodiol is then converted into catechol by a dehydrogenase enzyme. The catechol is then cleaved by intradiol dioxygenases or extradiol dioxygenases via ortho-cleavage or meta-cleavage pathways (Satpati et al. 2023; Hoque et al. 2023). In the ortho-pathway the ring of catechol is cleaved at the ortho-position by the intradiol dioxygenase enzyme such as 1,2-dioxygenase to form cis,cis-muconate. While, in the meta-cleavage pathway the catechol is cleaved by extradiol dioxygenases catechol 2,3-dioxygenase, to produce 2-hydroxymuconic semialdehyde (Patel et al. 2020; Tomar and Jajoo 2021). Both resulting intermediates cis,cis-muconate and 2-hydroxymuconic semialdehyde then enter into the tricarboxylic acid (TCA) cycle were they are further metabolized and ultimately leading to the complete oxidation of the aromatic ring (Nagy et al. 2024; Othman et al. 2023). Żyszka-Haberecht et al. (2019) reported the catabolic degradation of naphthalene. Naphthalene was broken down into cis-naphthalene dihydrodiol by naphthalene dioxygenase. The cis-naphthalene dihydrodiol was further oxidized to 1,2-dihydroxynaphthalene via dehydrogenation by dehydrogenase, which was then metabolized into 2-hydroxychromene 2-carboxylate by ring-opening reaction mediated by dioxygenase. The final products were further oxidized into compounds such as acetyl-CoA and succinyl-CoA which entered the tricarboxylic acid cycle. Similarly, Subashchandrabose et al. (2013) reported the oxidation of benzo(a)-pyrene by *Selenastrum capricornutum*, via dioxygenase enzyme system to cis-dihydrodiols which were then converted to sulphate ester as well as α and β-glucoside conjugates. Patel et al. (2020) reported the oxidation of phenanthrene which resulted in formation of cis-3,4-dihydroxy-3,4-dihydrophenanthrene by phenanthrene dioxygenase through hydroxylation. Then the cis-3,4-dihydroxy-3,4-dihydrophenanthrene was broken down into 3,4-dihydroxyphenanthrene after undergoing dehydrogenation. This is afterwards metabolized to 1-hydroxy 2-naphthoic acid and oxidized via salicylaldehyde and salicylic acid to catechol.

## Spent oil waste management

### Challenges associated with spent oil waste management

Over the years due to frequent environmental pollution in the world, the oil-based product industries have been in search of new formulation to produced biodegradable lubricant oil (Gunningham et al. 2019). On the other hand, SOW management scheme in several forms is present in different nations, but no standard or best practices exist. According to Gunningham et al. (2019), the industrial sector is experiencing a rise in operational costs due to high energy consumption. Managing the collection and disposal of SOW poses significant challenges such as financial strains, land-use constraints and insufficient infrastructure. The collection, transportation, disposal and treatment of SOW requires huge capital investment. For instance, the cost of a SOW collection truck ranges from $150,000 to $350,000 and operational cost for each truck vary from $30,000 to $50,000 per year, including fuel, repairs, and labour (Hopewell et al. 2020). The cost of transporting SOW to a disposal facility can make up 20 to 100% of the overall operational cost. In developed countries, this could range from $20 to $100 per ton of SOW transported, depending on the distance and environmental regulatory requirements (Hopewell et al. 2020). The cost of disposal of SOW at specialized facilities, such as incinerators, ranges from $200 to $500 per ton due to strict environmental regulations (Verma et al. 2021). The cost of constructing a SOW disposal facility that complies with environmental regulations requires an average capital of $10–20 million with annual maintenance costs of $100,000–$200,000 for medium to large scale industries (ICIS 2018). Financing and training the employees (drivers, operators, and technicians), paying regulatory compliance fees and insurance result in an additional annual operational cost by of $1–3 million (ICIS 2018). These costs underscore the financial challenge faced by industries in managing SOW effectively. Again, once the SOW is disposed off inappropriately, the risk of environmental pollution is inevitable (Kajdas 2014; Ngene et al. 2016).

### Legislation and regulations on spent oil waste management

Laws and regulations form a substantial part in the management of SOW. Different nations have their laws and regulatory policies governing the collection and disposal of SOW in accordance with local needs and environmental hazards (Kajdas 2014). For example, Resource Conservation and Recovery Act (RCRA) in the USA, and European Union (EU) Waste Framework Directive (2008/98/EC) states that SOW is classified as hazardous waste and environmentally unfriendly (Johansson 2023; Ozymy and Ozymy 2022). The RCRA and EU Waste Framework Directive further stated that it requires licensed facilities, industrialists, and manufacturers to collect SOW in an airtight container, transport it to licensed facilities and treat it to mitigate environmental impacts (Johansson 2023; Ozymy and Ozymy 2022). South Africa’s National Environmental Management: Waste Act (Act No. 59 of 2008) orders the responsible handling and disposal of SOW as well as emphasizes the need for industrialist to ensure sustainable waste management approach (Nyika et al. 2020). These laws from the different countries have been reported to significantly reduced SOW pollution with strict regulations on its handling, collection, transportation, and treatment (Nyika et al. 2020). By classifying SOW as hazardous waste, the RCRA in the USA, the EU Waste Framework Directive, and South Africa’s National Environmental Management: Waste Act (Act No. 59 of 2008) ensure that industries and manufacturers adhere to sustainable SOW management practices. The requirement for licensed facilities to process SOW prevents improper disposal, reducing soil and water contamination. Likewise, these regulations encourage recycling and recovery initiatives, minimizing the environmental impact of SOW (Nyika et al. 2020). Many countries also align their waste regulations with international waste act to ensure compliance with global environmental standards. The situation is different in some countries including Kenya, Nigeria, Zimbabwe, India, Benin, Togo, Mozambique, Namibia and Uganda where there is no strict legislation, lack of regulatory policy, supervision, and implementation of an effective modern waste management plan for the collection and disposal of SOW (Kajdas 2014). The government in collaboration with industrial sectors and private sectors needs to adopt an all-round strategy for the proper implementation of the Waste Act. Through the proper legal implementation of the Waste Act, the SOW industrial sectors could ensure proper compliance.

### Challenges, limitations, and strategies to improve microalgae-based biodegradation of SOW

Microalgae-based biodegradation offer a sustainable eco-friendly strategy for treating SOW. This approach faces several challenges and limitations. One major challenge is the low bioavailability of SOW, which limits microalgae ability to absorb and metabolize these pollutants effectively, in turn delays the overall degradation process (Calatrava et al. 2024). High concentrations of some SOW pollutants, particularly the aromatic HCs, can be toxic to microalgae, inhibiting their growth and metabolic activity (Ali et al. 2024). Another limitation is the slow degradation rate, as microalgae primarily use enzymatic pathways for the biotransformation of SOW (Calatrava et al. 2024). This slow degradation rate limits the efficacy of microalgae as a rapid response solution to environmental pollution. Environmental factors such as light, temperature, pH, and nutrient availability can significantly influence microalgal performance in SOW polluted environments and maintaining optimal conditions can be very challenging (Calatrava et al. 2024; Kamyab et al. 2017). Efficiently harvesting microalgae biomass from treated oil waste medium remain a technical challenge (Ali et al. 2024).

To overcome these challenges, screening and isolating hydrocarbon-degrading microalgae can enhance SOW biodegradation. Genetic engineering techniques can be employed to create microalgae strains with enhanced degradative capabilities, thereby improving their efficiency in breaking down SOW pollutants (Eregie et al. 2023). Co-cultivation with other hydrocarbon-degrading microorganisms could improve degradation efficiency via synergistic interactions and enhanced bioavailability of SOW pollutants (Ali et al. 2024). Optimizing the cultivation and growth conditions of microalgae such as light, temperature, pH, and nutrients (Ngerem et al. 2025; Kamyab et al. 2018) can substantially enhance microalgal tolerance to SOW towards improved biodegradation efficiency (Ali et al. 2024). The use of immobilization techniques can protect microalgae from toxic SOW pollutants and improve biomass recovery while the use of bioreactors could be employed to control degradation conditions leading to enhanced oil degradation (Calatrava et al. 2024).

## Conclusion and future prospect

Environmental pollution caused by SOW has attracted great concern due to its adverse impacts. The use of microalgae to remove SOW contaminants from the environment is a promising alternative to reduce costs and decrease environmental impacts. Despite the increasing interest in microalgae-based treatment for pollution control, its application remains relatively limited. Microalgae utilize oil pollutants as carbon sources for growth and metabolic activity, converting them into valuable products through redox reactions facilitated by catalytic enzymes. Given its sustainability and effectiveness, microalgae-based technology shows significant potential for SOW bioremediation. Future research should focus on optimizing microalgal strains for enhanced degradation efficiency, scaling up bioremediation systems, and integrating omics approaches to determine the catabolic genes and enzymes as well as metabolic pathways involved in SOW pollutant breakdown. These advancements are crucial for the development of innovative, biobased solutions for environmental pollution remediation.

Using bibliometric analysis carried out on 7,785 articles on SOW contaminant remediation indicated a remarkable increase (80.23%) in the number of published articles from 2005 to 2023. Environmental sciences with 3,571 publications were the most productive subject areas in this research field. While Bioresource Technology (249 publications) was the most prolific journals in this research field. China emerged as the top contributors with 887 publications. Ruan, R. from the University of Minnesota Twin Cities, USA was the most prolific author (39 publications), while the Harbin Institute of Technology, China was the most productive organization with 72 publications in this research area. The bibliometric analysis reveals the critical need for collaborative biodegradation effort to improve microalgae-based SOW degradation approach towards environmental sustainability. This could further accelerate research innovation and grant access to advance technologies in SOW contaminant remediation studies.

## Data Availability

No datasets were generated or analysed during the current study.

## Abbreviations

SOW: Spent oil waste
HCs: Hydrocarbon compounds
USA: United State of America
UK: United Kingdom
EU: European Union
PCB: Polychlorinated biphenyls
TCA: Tricarboxylic acid cycle
PAHs: Polycyclic aromatic hydrocarbons
BTEX: Benzene, toluene, ethylbenzene, and xylenes
RCRA: Resource conservation and recovery act
